# Advanced multifunctional nano-delivery platform focusing on treating diseases related to lipid metabolism via targeted intervention in various lipid metabolic processes

**DOI:** 10.1186/s40779-025-00672-6

**Published:** 2025-12-04

**Authors:** Ying Sun, Kai Yan, Yi Zhang, Yan-Qi Han, Long-Hui Hao, Yue Gao, Hong-Liang Wang, Hong-Qian Chu, Jun Ye, Yu-Ling Liu, Yan-Fang Yang

**Affiliations:** 1 State Key Laboratory of Bioactive Substance and Function of Natural Medicines, Institute of Materia Medica, Chinese Academy of Medical Sciences & Peking Union Medical College, Beijing, 100050 China; 2 Beijing Key Laboratory of Drug Delivery Technology and Novel Formulation, Institute of Materia Medica, Chinese Academy of Medical Sciences & Peking Union Medical College, Beijing, 100050 China; 3 Children Hospital of Fudan University, Shanghai, 201102 China; 4 Translational Medicine Center, Beijing Chest Hospital, Capital Medical University, Beijing, 101149 China

**Keywords:** Multifunctional nano-delivery system, Lipid metabolism, Lipid metabolism reprogramming, Lipid metabolism-related diseases

## Abstract

Disruptions in lipid metabolism cause numerous metabolic diseases, including obesity, diabetes, cardiovascular diseases, and liver disorders. Consequently, lipid metabolism serves as a potential therapeutic target, influencing the progression of various non-metabolic diseases such as kidney diseases, cancer, neurodegenerative disorders, aging, and bone-related diseases. The metabolic pathways involved in lipid metabolism are complex and highly interconnected. Although the abundance of metabolic targets presents opportunities for lipid metabolism regulation, the limited precision and safety of traditional therapeutic approaches remain significant challenges. These limitations have catalyzed the development of multifunctional nano-delivery platforms aimed at targeted intervention in lipid metabolic processes, further enhancing the flexibility of lipid metabolism regulation. This review outlines the latest advancements and representative applications of these multifunctional nano-delivery platforms. Notably, extensive research has been conducted on nanoparticles and liposomes, with these technologies being relatively mature. Furthermore, numerous novel biomaterials, including engineered adipocytes, exosome vesicles secreted by natural cells, smart-responsive nanomicelles, composite hydrogels, and engineered lipid droplets, are being increasingly explored. Finally, the review discusses the advantages of drug delivery strategies based on the targeted intervention of lipid metabolic processes, the limitations of current technologies, promising future research directions, and treatment challenges.

## Background

Lipids play essential roles in cellular and physiological processes and are ubiquitously distributed across animals, plants, and microorganisms. Based on structural features and biosynthetic origins, they can be broadly categorized into 8 major classes: fatty acids (FAs), sterols, glycerolipids, glycerophospholipids, sphingolipids, prenols, saccharolipids, and polyketides [1]. Among them, FAs represent the simplest type of lipids and serve as fundamental building blocks of more complex lipid molecules. Meanwhile, cholesterol, the prototypical sterol, is an indispensable component of eukaryotic cell membranes and a key precursor for diverse signaling molecules and metabolic products [2–5].

Lipid metabolism refers to a series of biochemical processes involving uptake, synthesis, lipolysis, storage, oxidation, and metabolic reprogramming, which form the basis of energy storage and supply and play indispensable roles in cell membrane architecture, hormone biosynthesis, immune modulation, neural function, and thermoregulation [6]. Subsequently, disruption and dysregulation of lipid metabolism are the underlying causes of various metabolic diseases, including obesity, diabetes, cardiovascular diseases (CVDs), and liver disorders. Additionally, lipid metabolism acts as a potential therapeutic target as it influences the progression of non-metabolic diseases, such as kidney disease, cancer, neurodegenerative disorders, aging, and bone-related diseases [7]. This highly coordinated network is dependent on: 1) the coordination and division of labor among multiple organs and cell types, such as the involvement of the liver in lipid synthesis, β-oxidation, the conversion of cholesterol to bile acids, the role of adipose tissue in triglyceride (TG) storage and the thermogenesis of brown adipocytes [8]; 2) a multi-layered regulatory network, including transcriptional regulation by sterol regulatory element-binding protein (SREBP) and peroxisome proliferator-activated receptors (PPARs); post-translational modifications by adenosine monophosphate-activated protein kinase (AMPK) and glucagon; and epigenetic regulation via histone modifications [9, 10]; and 3) the maintenance of dynamic balance, where insulin promotes lipogenesis, and adrenaline stimulates lipolysis, and lipid metabolism flexibly switches between synthesis, storage, and energy supply [11]. Thus, lipid metabolism is a highly complex series of processes that requires precise regulation. Any disruption of a single process or organelle may trigger a domino effect, leading to a series of physiological alterations and ultimately resulting in pathological conditions.

Currently, several drugs targeting lipid metabolism pathways are used to treat lipid metabolism disorders, such as Inclisiran (Food and Drug Administration-approved) for hypercholesterolemia, and Volanesorsen for familial chylomicronemia syndrome (FCS) [12, 13]. Inclisiran is a small interfering RNA (siRNA) that regulates lipid metabolism by inhibiting the synthesis of proprotein convertase subtilisin/kexin type 9 (PCSK9), while Volanesorsen is an antisense oligonucleotide that regulates lipid metabolism by inhibiting the synthesis of apolipoprotein C-III. However, both agents present notable limitations that hinder their broader application. While Inclisiran primarily targets the liver with limited tissue specificity, and its injectable administration reduces patient compliance, Volanesorsen is associated with a high risk of thrombocytopenia and is restricted to patients with FCS harboring specific genetic mutations [14, 15]. Therefore, further investigation into precise targets within lipid metabolism processes and advanced drug delivery systems is meaningful and essential for the development of highly targeted drugs with clear mechanisms, strong efficacy, high safety, and improved patient compliance. Such drugs could address the shortcomings of traditional treatments, significantly enhancing the targeting accuracy and therapeutic effectiveness of lipid metabolism interventions.

Compared to traditional systemic drug administration, nano-delivery systems targeting lipid metabolism processes offer significant advantages. In terms of both efficacy and safety, targeted drug accumulation in specific tissues reduces systemic exposure while optimizing dosage, which may improve patient adherence [16]. For instance, nucleic acid drugs are prone to enzymatic degradation; however, nanocarriers can stabilize them and facilitate cellular uptake to overcome bioavailability limitations [17]. Additionally, nanocarriers can be designed to respond intelligently to microenvironmental cues or be controlled in a spatiotemporal manner, allowing for precise regulation of selected targets or pathways. This approach minimizes unintended interference with other lipid metabolism pathways, reducing side effects [18]. Carriers can also integrate imaging agents and therapeutic drugs to enable real-time monitoring of treatment efficacy, achieving integrated diagnostic and therapeutic outcomes [19]. Moreover, considering the unique properties of lipid metabolism, which contains diverse molecular species, organ cooperation, and multilayered regulatory networks, systems can be designed to co-deliver lipid metabolism inhibitors and small-molecule drugs to achieve synergistic therapeutic effects. Alternatively, combined delivery of gene editing tools and lipid metabolism inhibitors enables dual-level control over gene expression and lipid metabolic processes [20].

**Fig. 1:**
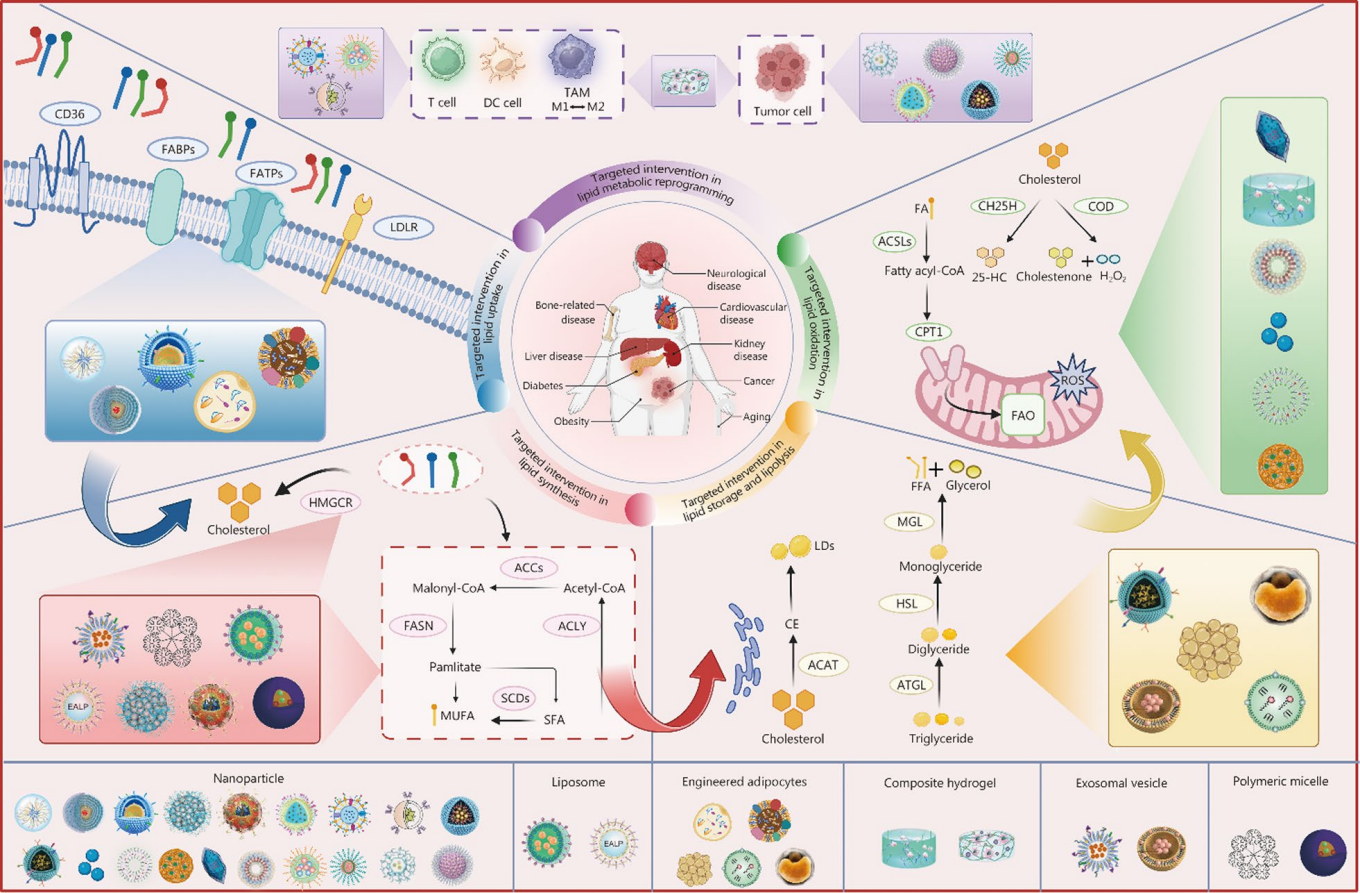
The overview scheme. Lipid metabolism is a highly complex and multifaceted process, consisting of lipid uptake, lipid synthesis, lipid storage, lipolysis, and lipid oxidation, as well as metabolic reprogramming in pathological states. These processes include multiple key enzymes, transport proteins, and receptors, which serve as necessary regulatory nodes. The link between lipid metabolism and disease is widely acknowledged as a crucial biological determinant. Nano-delivery platforms engineered to regulate lipid metabolic processes have exhibited a remarkable capacity to traverse complicated metabolic landscapes and precisely target key regulatory sites, thereby reinstating metabolic balance. This image is created with BioRender. CD36 cluster of differentiation 36, FABPs fatty acid-binding proteins, FATPs fatty acid transport proteins, LDLR low-density lipoprotein receptor, DC dendritic cell, TAM tumor-associated macrophage, M1 pro-inflammatory phenotype, M2 anti-inflammatory phenotypes, HMGCR 3-hydroxy-3-methylglutaryl (HMG)-CoA reductase, ROS reactive oxygen species, EALP a tumor-penetrating nanovesicle system namely EALP, ACC acetyl-CoA carboxylase, SCDs stearoyl-CoA desaturase, MUFA monounsaturated fatty acid, SFA saturated fatty acid, CE cholesteryl ester, LDs lipid droplets, ACAT acyl coenzyme A-cholesterol acyltransferase, FFA free fatty acid, MGL monoglyceride lipase, HSL hormone-sensitive lipase, ATGL adipose triglyceride lipase, FAO fatty acid β-oxidation, CPT1 carnitine palmitoyl transferase, FA fatty acid, ACSL acyl-CoA synthetase, COD cholesterol oxidase, CH25H cholesterol 25-hydroxylase, 25-HC 25-hydroxycholesterol

In this review, we aimed to provide an overview of the conventional processes of lipid (FAs and cholesterol) metabolism-uptake, synthesis, storage, lipolysis, oxidation, and metabolic reprogramming under abnormal physiological conditions. We highlighted the key enzymes and pathways involved in each of these processes. Additionally, we examined the role of lipid metabolism as a primary and secondary factor in the pathogenesis of various diseases, analyzing the regulatory functions of key enzymes and pathways introduced above, and discussing current pharmacological strategies targeting lipid metabolism, along with their respective advantages and limitations. Subsequently, we reviewed the latest advancements and representative applications on multifunctional nano-delivery platforms for lipid metabolism-targeted interventions (Fig. 1), including nanoparticles (NPs), liposomes, stimuli-responsive polymeric micelles, and a range of biomimetic materials that have recently emerged, including modified adipocytes, naturally secreted exosomal vesicles, and engineered lipid droplets (LDs). Finally, the advantages of drug delivery strategies based on the targeted intervention of lipid metabolic processes, the limitations of current technologies, promising future research directions, and challenges are discussed.

## Process of lipid metabolism

**Fig. 2:**
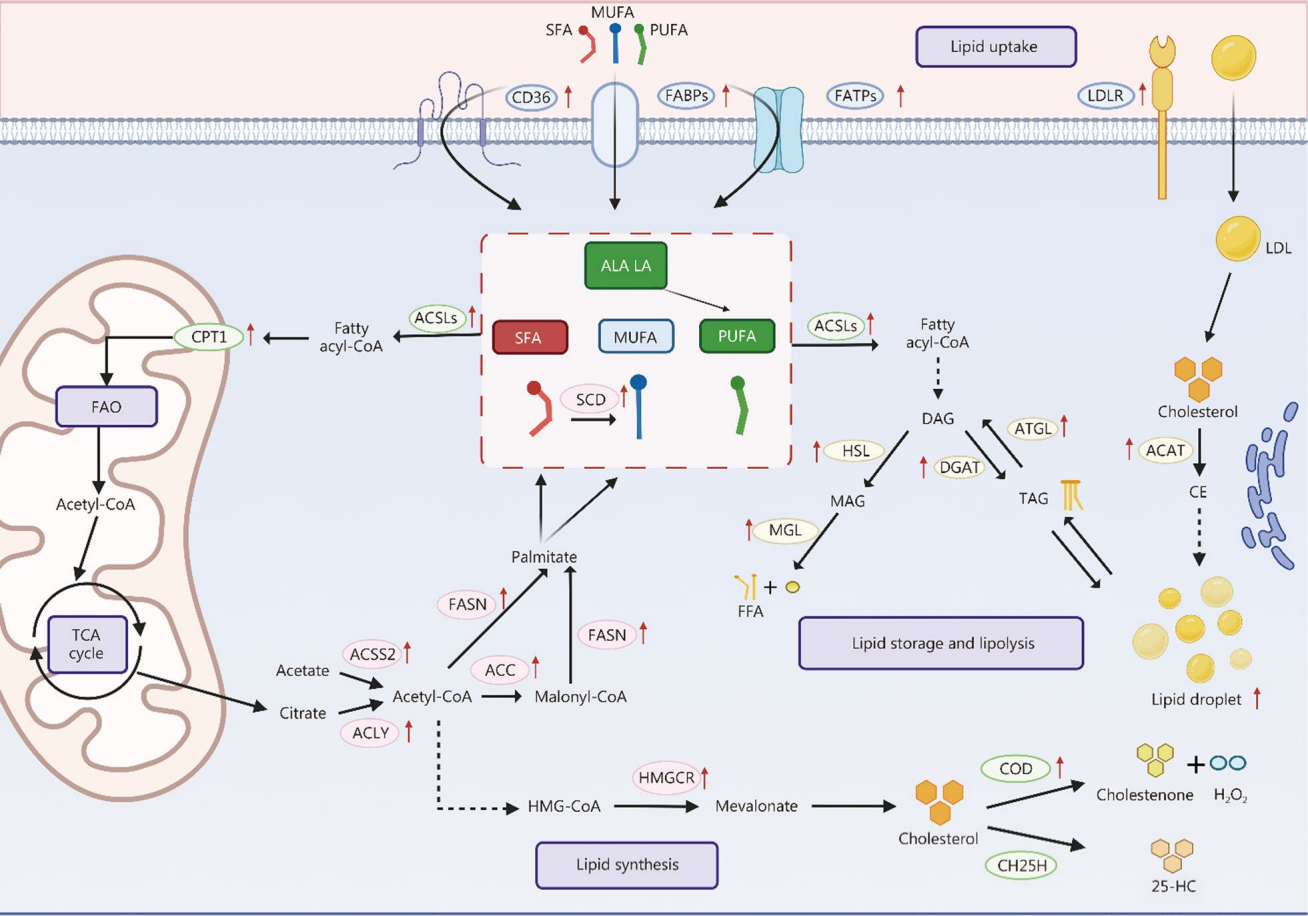
The main processes of lipid metabolism and key target-related changes induced by lipid metabolic reprogramming. Lipid metabolism is fundamentally divided into 4 principal pathways: lipid uptake, lipid synthesis, lipid storage and lipolysis, and lipid oxidation. Key targets involved in lipid uptake include CD36, FABPs, FATPs, and LDLR; Key targets regulating lipid synthesis include ACLY, ACSS, ACC, FASN, SCD, and HMGCR; Key targets responsible for lipid storage and lipolysis include DAGT, ATGL, HSL, and MGLL; Key targets governing lipid oxidation include ACSLs, CPT1, CPT2, and COD. Lipid metabolic reprogramming manifests primarily as the upregulation of these metabolic targets, resulting in the accumulation of both endogenous and exogenous lipid pools and an overall intensification of lipid metabolic activity. This image is created with BioRender. CD36 cluster of differentiation 36, FABPs fatty acid-binding proteins, FATPs fatty acid transport proteins, LDLR low-density lipoprotein receptor, CPT carnitine palmitoyl transferase, FAO fatty acid β-oxidation, CoA coenzyme A, TCA tricarboxylic acid, ACSLs acyl-CoA synthetase, SFA saturated fatty acid, ALA alpha-linolenic acid, LA linoleic acid, MUFA monounsaturated fatty acid, PUFA polyunsaturated fatty acid, SCD stearoyl-CoA desaturase, LDL low-density lipoprotein, HSL hormone-sensitive lipase, DAG diacylglycerol, MAG monoacylglycerol, TAG triacylglycerol, ATGL adipose triglyceride lipase, MGL monoglyceride lipase, DGAT diacylglycerol acyltransferase, ACAT acyl coenzyme A-cholesterol acyltransferase, CE cholesteryl ester, FFA free fatty acid, FASN fatty acid synthase, ACSS acetyl-CoA by acetyl-CoA synthetase, ACC acetyl-CoA carboxylase, ACYL ATP-citrate lyase, HMG 3-hydroxy-3-methylglutaryl, HMGCR 3-hydroxy-3-methylglutaryl (HMG)-CoA reductase, COD cholesterol oxidase, CH25H cholesterol 25-hydroxylase, 25-HC 25-hydroxycholesterol

Molecules such as FAs, cholesterol, and TG are closely involved with multiple essential organelles in lipid metabolism. For example, the endoplasmic reticulum (ER) is involved in FA synthesis, lipid storage, and transport; the Golgi apparatus is responsible for lipid processing and trafficking; mitochondria are the primary site for β-oxidation of FAs; the cell membrane is closely associated with lipid distribution and transport; and lysosomes are involved in lipid degradation [21]. Disruptions in any metabolic process or organelle can result in pathological conditions, under which cells adjust their energy demands to adapt to pathological conditions. Specifically, cells undergo metabolic adaptations to cope with external environmental stress, enabling them to acquire new functional properties. This metabolic flexibility is closely linked to lipid metabolic reprogramming, which plays a crucial role in maintaining cellular homeostasis and responding to environmental challenges [22]. This section discusses the complete metabolic processes of 2 major types of lipids, FAs and cholesterol, including their uptake, synthesis, storage, lipolysis, and oxidation under normal physiological conditions. Moreover, it examines common target alterations and recent research advancements in these processes within the landscape of lipid metabolic reprogramming, providing a foundation for the treatment of lipid metabolism-related diseases and the design of drug delivery carriers (Fig. 2).

### Lipid uptake

#### FA uptake

Extracellular and intracellular lipids constitute 2 major sources of FAs. Circulating free FAs (FFAs) in the bloodstream are primarily taken up via FA transporters located in the plasma membrane. Among them, cluster of differentiation 36 (CD36), FA-binding proteins (FABPs), and fatty acid transport proteins (FATPs) are the most extensively studied FA transporters [23, 24]. The known intracellular sources of FAs include lipoproteins and macropinocytosis, while LDs generate FFAs through lysosomal hydrolysis [25].

CD36-mediated FAs uptake can be divided into 2 sequential stages. Initially, CD36 binds to extracellular FAs and facilitates their insertion into the outer leaflet of the lipid bilayer; Subsequently, FAs undergo transbilayer “flip-flop” diffusion to the inner leaflet and associate with intracellular FABPs to complete transport. Once inside the cell, FAs bind to FABPs and other protein transporters, working in conjunction with CD36 to facilitate subsequent transport steps [26]. Definitive studies indicate that CD36 facilitates the transport of nearly half of the FAs in mouse adipose tissue and muscle [27]. In addition, CD36 plays a crucial role in enhancing FAs uptake in the heart, skeletal muscle, and adipose tissues [28, 29]. In recent years, researchers have continued to investigate the complex mechanisms underlying CD36-mediated FAs transport. A recent study have identified 2 palmitoyltransferases-Asp-His-His-Cys (DHHC) 4, localized in the Golgi apparatus, and DHHC5, positioned on the plasma membrane, as regulators of CD36-mediated FAs uptake. This finding has broadened the scope of protein palmitoylation studies, extending them into the field of metabolic biology [30]. Consistent with this finding, Hao et al. [31] revealed that FAs binding to CD36 activates the downstream Lck/Yes-related novel tyrosine kinase, which phosphorylates and inactivates DHHC5, leading to CD36 depalmitoylation. This modification triggers CD36 endocytosis, thereby promoting FAs uptake into the cell. Beyond facilitating FAs transmembrane transport, CD36 also serves as a regulator of the FAs uptake rate. Bonen et al. [32] established a skeletal muscle contraction model, demonstrating that muscle contraction induces CD36 translocation, where it covalently binds to a long-chain FA (LCFA) derivative, thereby attenuating the rate of contraction-induced FAs uptake.

FAs are lipophilic molecules with limited solubility in aqueous environments within the human body. Therefore, some studies suggest that FAs undergo passive diffusion across membranes owing to their limited water solubility [33–35]; however, the efficiency of this process largely depends on the cooperative action of FABPs and FATPs [23]. Among them, plasma membrane-associated FABP (FABPpm), which is localized to the exterior of the plasma membrane, interacts with CD36 to facilitate the initial uptake of FAs, whereas cytoplasmic FABP (FABPc), which resides within the cytosol, mediates FAs binding once they enter the aqueous cytosolic phase. This desorption step represents the rate-limiting event of FAs transport [36, 37]. FATPs are ubiquitously expressed across multiple tissues and exhibit dual functions of transport and enzymatic activity. They catalyze FAs activation into acyl-CoA and preferentially channel these intermediates toward TG synthesis, thereby acting as critical determinants of lipid deposition and trafficking [38]. Additionally, recent studies suggest that FATP-mediated FAs transport may be linked to mitochondrial function [39, 40]. For example, Miner et al. [41] identified perilipin 5 as a key mediator of LDs-mitochondria contact sites. At these contact points, it interacts with FATP4, facilitating FAs transfer from LDs to mitochondria.

#### Cholesterol uptake

Cholesterol balance is maintained through 2 primary pathways: endogenous cholesterol biosynthesis and exogenous cholesterol uptake. Dietary cholesterol absorption is regulated by Niemann-Pick type C1-like 1 (NPC1L1)-mediated intestinal uptake and low-density lipoprotein receptor (LDLR)-mediated low-density lipoprotein-cholesterol (LDL-C) uptake. In intestinal epithelial cells, NPC1L1 mediates cholesterol uptake from the intestinal lumen into enterocytes. Once inside, cholesterol translocates from the plasma membrane to the ER, where it is esterified by acyl coenzyme A-cholesterol acyltransferase 2 (ACAT2, also known as sterol O-acyltransferase, SOAT2) into cholesteryl esters (CEs).

Previous studies have shown that under cholesterol depletion, NPC1L1 translocates from the endocytic recycling compartment (ERC) to the plasma membrane, while cholesterol binding triggers its clathrin and adaptor protein 2-dependent endocytosis to the ERC [42, 43]. There, cholesterol is transferred to the ER for esterification, and NPC1L1 subsequently recycles to the membrane. Ferrari et al. [44] explored the mechanism of non-vesicular cholesterol transport to the ER and identified Aster as a non-vesicular cholesterol transporter. Their experiments proved in wild-type mice, cholesterol binding to NPC1L1 on intestinal epithelial cells triggers the recruitment of Aster proteins to the plasma membrane. These proteins establish contact sites between the plasma membrane and the ER, enabling the direct transfer of cholesterol to the ER, where ACAT2 catalyzes its esterification into CEs. Subsequently, these CEs, together with apolipoproteins and TGs, are assembled into chylomicrons (CMs) and then secreted into systemic circulation [45]. Conversely, in Aster-deficient mice, cholesterol accumulates in the intestinal cell plasma membrane, while the ER exhibits cholesterol depletion. This condition is characterized by decreased CEs storage and upregulation of the SREBP-2 transcriptional pathway.

As the primary organ for cholesterol synthesis, the liver releases endogenous and exogenous cholesterol into circulation in the form of very-low-density lipoproteins (VLDLs), which are converted into LDL for uptake by LDLRs on peripheral cells. Meanwhile, excess cholesterol in peripheral tissues is returned to the liver via reverse cholesterol transport in the form of high-density lipoprotein cholesterol (HDL-C), where it is either recycled or excreted as bile acids. Studies have shown that the Really Interesting New Gene E3 ubiquitin ligase inducible degrader of the LDL receptor (IDOL, also known as Myosin Regulatory Light Chain Interacting Protein, MYLIP) and PCSK9 promote the induced degradation of LDLR, thereby inhibiting cholesterol uptake [46]. After LDLR is recognized by IDOL and undergoes ubiquitination, it is internalized by the endocytic adaptor epsin 1 and sorted into the lysosome for degradation [47]. Meanwhile, following post-translational modifications in the cell, PCSK9 binds to the extracellular domain of LDLR, forming a PCSK9-LDLR complex, which is internalized via clathrin-coated pits and finally directed to lysosomes for degradation [48].

### Lipid synthesis

#### FAs synthesis

In addition to exogenous lipid uptake, intracellular lipid accumulation can occur via endogenous biosynthesis, a process tightly regulated by key enzymes in lipid metabolic pathways. This phenomenon, termed de novo lipogenesis (DNL), plays an important role in lipid balance and has long been recognized as a hallmark of cancer. Up to 90% of lipids are synthesized via this pathway in tumor cells, underscoring its role in lipid metabolic reprogramming (see Section "Lipid metabolic reprogramming" for further discussion) [49, 50]. Lipid biosynthesis predominantly occurs in the liver and adipose tissue, utilizing acetyl-CoA as the central substrate. The initial sources of acetyl-CoA originate from FA β-oxidation (FAO) and glycolysis, where nutrients are metabolized through the tricarboxylic acid (TCA) cycle in mitochondria to generate citrate [51, 52]. Mitochondrial citrate is then exported to the cytosol via mitochondrial citrate transport proteins, where it is cleaved by ATP-citrate lyase (ACLY) to regenerate acetyl-CoA, fueling lipid biosynthesis. In hypoxic cancer cells, glycolysis suppression leads to a reduction in TCA cycle-derived citrate levels. Subsequently, acetate serves as an alternative carbon source, which is converted into acetyl-CoA by acetyl-CoA synthetase (ACSS), constituting an alternative pathway for acetyl-CoA production during DNL [53]. The regenerated acetyl-CoA serves as a precursor for 2 major metabolic pathways: FAs synthesis and cholesterol biosynthesis. In the FAs synthesis pathway, acetyl-CoA is first carboxylated to malonyl-CoA by acetyl-CoA carboxylase (ACC). This is followed by a series of condensation reactions catalyzed by FAs synthase (FASN), leading to the formation of palmitic acid (PA) (C16:0), the most abundant saturated fatty acid (SFAs) in the human body. This process is highly dependent on nicotinamide adenine dinucleotide phosphate hydrogen (NADPH) as a reducing equivalent donor [54, 55]. PA can be further elongated and desaturated by stearoyl-CoA desaturase (SCD) to produce monounsaturated fatty acids (MUFAs), which are essential for various physiological processes [56].

##### ATP-citrate lyase (ACLY)

ACLY cleaves citrate, which is transported from the mitochondrial matrix, and serves as the rate-limiting enzyme for acetyl-CoA regeneration, marking the first step of lipid synthesis. Studies have shown that the expression and activity of ACLY are regulated by multiple factors. Lin et al. [57] discovered that under high glucose conditions, ACLY is acetylated by P300/calcium-binding protein-associated factor acetyltransferase at lysine residues K540, K546, and K554, which prevents ubiquitination and thereby enhances its stability. Additionally, Migita et al. [58] elucidated the role of phosphorylation in enhancing ACLY activity. They reported that the phosphorylation status of ACLY in the A549 cell line decreased in a dose and time-dependent manner in the presence of a phosphoinositide 3-kinase (PI3K) inhibitor. The downstream effector Akt (also known as protein kinase B, PKB) exhibited a similar regulatory effect, thereby confirming that the PI3K/Akt pathway regulates ACLY phosphorylation. In addition to its role in phosphorylation regulation, Akt stimulates ACLY-mediated acetylation. Moreover, Akt activates ACLY phosphorylation, ensuring the maintenance of histone acetylation levels, even in low glucose environments [59]. Study has also illustrated that under cold conditions, the expression of ACLY increases in brown adipose tissue, promoting FAO, which aids in maintaining body temperature [60].

##### Acetyl-CoA synthetase (ACSS)

In tumor cells deprived of blood, oxygen, and nutrients, ACSS enhances the utilization of exogenous free acetate from plasma and interstitial fluid, using acetate as an additional nutrient source to synthesize acetyl-CoA, which enables tumor cells to adapt to a harsh metabolic environment [54]. Reportedly, at least 3 types of ACSS exist in mammals. ACSS1 and ACSS2 preferentially use acetate as their substrate, while ACSS3 has a higher affinity for propionate [61]. Among them, ACSS1 and ACSS3 are mitochondrial proteins, whereas ACSS2 is located in the cytoplasm and nucleus, and is expressed widely across various cell types under different physiological conditions [62]. Due to its efficient use of acetate, ACSS2 is considered a key enzyme in acetyl-CoA synthesis. Accordingly, a previous study reported that the upregulation and knockdown of ACSS2 in glioblastoma cells significantly impact tumor proliferation and suppression [63]. Research has also shown that the expression of ACSS2 is transcriptionally regulated by Specificity protein 1, a target of SREBPs, further confirming its role in lipid synthesis [64]. Additionally, ACSS2 regulates lipid metabolism by inducing changes in histone structure and promoting acetylation through acetate [65, 66].

##### Acetyl-CoA carboxylase (ACC)

ACC catalyzes the conversion of acetyl-CoA into malonyl-CoA, a key regulatory enzyme in FA synthesis. ACC1 (ACCα, 265 kD) and ACC2 (ACCβ, 275 kD) are the known forms of ACC. ACC1 is located in the cytosol, where it plays a primary role in malonyl-CoA production and the subsequent elongation of FAs to form long-chain polyunsaturated fatty acids (PUFAs). In contrast, ACC2 is located on the outer mitochondrial membrane. The malonyl-CoA generated by ACC2 locally allosterically inhibits carnitine palmitoyl transferase 1, which primarily negatively regulates FAO [67]. Although these isoforms are believed to have distinct roles, they catalyze the same biochemical reaction through similar enzymatic mechanisms. Additionally, ACC1 and ACC2 are downstream targets of AMPK regulation; therefore, their activities can be inhibited by AMPK-mediated phosphorylation. This inhibition decreases FA and cholesterol synthesis and promotes cellular lipid degradation [68]. Furthermore, the activity of ACC1 is regulated by domain conformational changes, protein–protein interactions, and transcriptional regulation by SREBPs [69–71].

##### FASN

FASN is the central enzyme in DNL, catalyzing the synthesis of PA, a long-chain SFA, from acetyl-CoA and malonyl-CoA as substrates, with NADPH serving as a continuous electron donor. The catalytic process of FASN involves 7 key steps, including condensation, reduction, and dehydration. Additionally, it is one of the most extensively studied enzymes in the context of cancer-related FA metabolism. Overexpression or hyperactivation of FASN is closely linked to the progression and prognosis of several cancers and diseases [72]. FASN regulation is complex, involving the activation of multiple signaling pathways such as PI3K/Akt/mammalian target of rapamycin (mTOR), Wnt/β-catenin, mitogen-activated protein kinase, epidermal growth factor receptor, and SREBP, along with post-translational modifications like ubiquitination, acetylation, and SUMOylation [73–77]. Wang et al. [73] demonstrated that MK8722 activates the upstream autophagy pathway PI3K/Akt/mTOR, downregulating FASN expression in a dose-dependent manner, and inhibits downstream autophagy by blocking the fusion of autophagosomes and lysosomes, leading to epithelial ovarian cancer cell death. Notably, this process highlights FASN as a new molecular target bridging the gap between lipid metabolism and autophagy. Lu et al. [74] found that FA-binding protein 5 interacts with FASN, positively regulating its expression through the ubiquitin–proteasome pathway, which activates the Wnt/β-catenin signaling pathway and promotes lipid metabolism, playing a critical role in the progression of pancreatic neuroendocrine tumors. Furthermore, Lan et al. [75] reported that the traditional Chinese medicine Si-Ni-San reduces FASN expression by activating AMPK, which inhibits the activity of the transcriptional coactivator p300 and transcription factor SREBP-1c, thereby decreasing lipid accumulation and impairing lipid metabolism through a novel mechanism.

##### Stearoyl-CoA desaturase (SCD)

SCD1 is the primary monodesaturase catalyzing the conversion of stearic acid and PA (which is synthesized by FASN during DNL) into the MUFAs oleic and palmitoleic acid, respectively [78]. Thus, SCD1 plays a critical role in maintaining the balance between SFAs and MUFAs, which is essential for cellular metabolism and lipid biosynthesis regulation. SCD1 inhibition results in SFA accumulation, which provokes ER stress and lipotoxicity, ultimately leading to apoptosis [79]. Concurrently, PUFA accumulation drives ferroptosis, positioning SCD1 as a critical regulator of this cell death pathway [80, 81]. At the metabolic level, hepatic SCD1 inhibition downregulates SREBP-1c while upregulating peroxisome PPARγ and fibroblast growth factor 21, thereby attenuating DNL [82]. In adipose tissue, SCD1 inhibition upregulates glucose transporter (GLUT) expression and enhances insulin sensitivity. In the skin, SCD1 deficiency activates AMPK-mediated ACC phosphorylation, enhancing fatty acid oxidation and reducing lipid accumulation [83]. Meanwhile, SCD1 overexpression in skeletal muscle elevates PPARδ and uncoupling protein 1 (UCP1), suppresses protein tyrosine phosphatase 1B, and improves metabolic performance and exercise capacity via the PI3K/Akt/mTOR pathway [84]. Collectively, these findings underscore that SCD1 functions as a pivotal regulator of lipid metabolism and as a multifaceted modulator of cell death, energy homeostasis, and tissue-specific metabolic regulation [85].

#### Cholesterol synthesis

Acetyl-CoA obtained by the abovementioned regeneration is also involved in the endogenous biosynthesis of cholesterol [86]. This process primarily occurs in the ER of hepatocytes. Initially, two molecules of acetyl-CoA are converted into acetoacetyl-CoA by acetyl-CoA acetyltransferase, providing a precursor for cholesterol biosynthesis. This intermediate is then catalyzed by 3-hydroxy-3-methylglutaryl-CoA (HMG-CoA) synthase to form HMG-CoA. The conversion of HMG-CoA to mevalonate, catalyzed by the key enzyme 3-hydroxy-3-methylglutaryl (HMG)-CoA reductase (HMGCR), constitutes the committed step and the rate-limiting step of the cholesterol biosynthetic pathway. Additionally, squalene epoxidase (SQLE), another rate-limiting enzyme, facilitates the oxidation of squalene to 2,3-oxidosqualene, a crucial intermediate required for cholesterol synthesis [87].

The cholesterol biosynthesis pathway is regulated by multiple key enzymes, including HMGCR and SQLE, whose transcription is primarily controlled by SREBP-2. Among them, HMGCR catalyzes the first rate-limiting step in the mevalonate pathway, making its regulatory process a major focus of scientific research. Recent studies indicate that the HMGCR promoter exhibits low cholesterol responsiveness, requiring high levels of SREBP-2 for activation [88, 89]. This mechanism ensures that cells prioritize LDLR-mediated exogenous cholesterol uptake, thereby avoiding unnecessary or excessive cholesterol synthesis. In contrast, the SQLE promoter is more cholesterol-responsive and highly sensitive to fluctuations in SREBP-2 levels, allowing SQLE expression to be rapidly upregulated in response to cholesterol depletion, ensuring sufficient cholesterol biosynthesis when needed [90]. Notably, in addition to SREBP-mediated regulation, HMGCR and SQLE are subject to other regulatory mechanisms. Lu et al. [91] uncovered the mechanism of cholesterol synthesis after feeding: following food intake, increased insulin and glucose levels activate mTOR Complex 1 via the PI3K/Akt signaling pathway, which subsequently directly phosphorylates USP20, stabilizing HMGCR, preventing its degradation, and enhancing cholesterol biosynthesis. Simultaneously, p53 suppresses SQLE expression under normal cholesterol conditions by directly binding to the SQLE promoter region, thereby reducing cholesterol synthesis in an SREBP-2-independent manner [92]. Although SQLE is a rate-limiting enzyme in cholesterol biosynthesis, excessive cholesterol levels can induce SQLE protein degradation, a process predominantly mediated by MARCHF6-dependent ubiquitination and regulated via the GSK3β and p53 signaling pathways [93].

### Lipid storage and lipolysis

Lipid storage enables organisms to conserve surplus energy as fat, ensuring long-term metabolic stability. In humans, LDs function as the primary lipid reservoirs, with a core of neutral lipids, predominantly TGs and CEs, encased within a phospholipid monolayer embedded with regulatory proteins. Thus, LDs function as dynamic lipid reserves, mobilizing stored FAs for cellular energy metabolism, and as cytoprotective structures that safeguard cells from lipotoxic stress [94, 95]. Based on previous studies, LDs biogenesis originates in the ER, where neutral lipids accumulate. Once the intracellular concentration of neutral lipids surpasses a threshold level, they begin to coalesce into lens-shaped structures on the luminal leaflet of the ER membrane. Facilitated by ER-resident scaffolding proteins, including fat storage-inducing transmembrane proteins and seipin, these structures progressively stabilize into nucleation sites, ultimately budding off from the ER to form mature, independent LDs [96–99]. LDs formation involves several key enzymes, including diacylgycerol acyltransferase (DGAT), which converts diacylglycerol (DAG) to TG, and ACAT, which converts free cholesterol to CEs [94, 95, 100]. Subsequently, intracellular and intercellular LDs trafficking occurs, which is essential to fulfill cellular biological functions. Han et al. [101] discovered that the LDs transporter can simultaneously bind to phosphatidic acid and phosphatidylinositol 4,5-bisphosphate on LDs surfaces, while also interacting with myosin heavy chain 10 (MYH10). This dual interaction anchors LDs to the cytoskeleton, allowing MYH10, a motor protein, to facilitate LDs transport from the ER to other organelles, such as mitochondria, where LDs participate in cellular metabolic activities.

Lipolysis is a fundamental metabolic process in which TGs, phospholipids, and sterols are enzymatically degraded into smaller bioactive molecules. This process is a crucial component of lipid metabolism, playing an indispensable role in energy supply, thermoregulation, and intercellular signaling [102]. Recently, the importance of lipolysis in metabolic regulation has garnered widespread attention. Lipolysis is broadly categorized into neutral and acidic lipolysis. Neutral lipolysis constitutes the major intracellular TG degradation pathway, involving three key enzymes: adipose triglyceride lipase (ATGL), hormone-sensitive lipase (HSL), and monoglyceride lipase (MGLL). These enzymes function in a stepwise manner during TG hydrolysis: ATGL catalyzes the initial hydrolysis of TG into diglycerides (DG), followed by HSL, which further hydrolyzes DG into monoglycerides (MG). Finally, MGLL hydrolyzes MG to yield FFAs and glycerol, completing the lipolytic cascade [102, 103]. Meanwhile, acidic lipolysis represents the lipid degradation process occurring within lysosomes, with lysosomal acid lipase serving as the principal catalytic enzyme [104–106]. Ding et al. [107] confirmed that glucose deprivation leads to an upregulation of ATGL activity. Mechanistically, glucose depletion reduces phosphatidylinositol (4,5)-bisphosphate levels in the Golgi apparatus, thereby disrupting the assembly of the CUL7-FBXW8 E3 ubiquitin ligase complex, a key regulator of ATGL degradation. This disruption ultimately stabilizes ATGL and enhances its activity, facilitating increased lipid mobilization. Furthermore, HSL is localized in the cytoplasm in its inactive state under basal conditions. Upon adipocyte stimulation, phosphorylation of HSL at the Ser563 residue induces its translocation to the LDs surface, where it initiates lipolytic activity [108, 109]. Studies on MGLL have revealed that a reduction in N6-methyladenosine modification levels enhances MGLL mRNA stability, extends its half-life, and ultimately elevates MGLL protein expression, thereby promoting lipolysis [110].

Lipid storage and lipolysis are tightly regulated and functionally interdependent processes, which are influenced by certain proteins. The Perilipin (PLIN) protein family comprises key LDs-associated proteins that are primarily involved in LDs formation, stabilization, lipid metabolism, and signal transduction [96]. Among the PLIN family proteins, PLIN2 is the sole isoform that is ubiquitously and constitutively expressed across multiple cell types. This protein plays a crucial role in maintaining LDs integrity by inhibiting autophagy. Conversely, its depletion enhances autophagy-driven TG breakdown, underscoring its fundamental role in regulating lipid storage and lipolysis [111]. Notably, the muscle circadian clock also impacts lipid metabolism, especially lipid storage and lipolysis. The muscle circadian rhythm promotes neutral lipid storage by brain and muscle arnt-like 1-dependent activation of DGAT2, while REV-ERBα suppresses key target genes to downregulate lipid degradation and protein turnover [112]. Moreover, studies have revealed that the rhythmic regulation of lipid storage and lipolysis is closely associated with TG metabolism [102, 113]. Thus, a comprehensive understanding of the dynamic interplay between lipid storage and lipolysis will provide critical insights into disease pathogenesis and fundamental physiological processes.

### Lipid oxidation

#### FAO

FAO occurs in the mitochondria, where it serves as a key energy-generating process via the breakdown of FAs. This process begins with the activation of LCFAs by long-chain acyl-CoA synthetase (ACSL), forming fatty acyl-CoA. Subsequently, fatty acyl-CoA is then sequentially converted into fatty acylcarnitine by carnitine palmitoyltransferase 1 (CPT1), transported across the mitochondrial membrane, and converted back into acyl-CoA by CPT2 [114]. This acyl-CoA is then progressively cleaved into acetyl-CoA, which enters the TCA cycle, leading to the production of ATP and NADPH. Beyond energy metabolism, FAO is also essential for sustaining NADPH balance, mitochondrial integrity, and overall cellular homeostasis [115]. However, hyperactivation of FAO has been implicated in cellular toxicity. For instance, in cancer cells, high expression of DGAT1 facilitates the conversion of excess fatty acyl-CoA into TGs, which are stored in LDs. Conversely, silencing DGAT1 expression markedly enhances FAO activity while concurrently elevating reactive oxygen species (ROS) levels, exacerbating oxidative stress and metabolic dysregulation [116].

In addition, the AMPK signaling pathway and the PPAR family, especially PPARα, play necessary regulatory roles in FAO. For example, AMPK can be activated by leptin as a signal for adequate fat storage in adipose tissue, leading to enhanced FAO energy expenditure [117]. Furthermore, a functional interplay exists between AMPK signaling and PPARs. Evidence suggests that suppression of the protein kinase A-AMPK axis leads to downregulation of PPARα, peroxisome proliferator- activated receptor gamma coactivator 1α (PGC-1α), and silent information regulator two 1 (SIRT1), ultimately inhibiting FAO [118].

#### Cholesterol oxidation

Cholesterol oxidation refers to the chemical reactions that cholesterol undergoes in the presence of free radicals, oxidants, or enzymes, leading to the formation of a series of oxidized derivatives. These oxidized cholesterol metabolites participate in various physiological regulatory processes, including cholesterol metabolism modulation, alterations in membrane fluidity and permeability, gene expression control, signal transduction, and immune function modulation [119]. Notably, although cholesterol exhibits a lower oxidation rate constant than PUFAs, its oxidized derivatives accumulate at higher levels within cell membranes compared to PUFA oxidation products. This paradox may be attributed to the preferential localization of cholesterol in lipid rafts, which are deficient in antioxidants such as vitamin E [120].

Cholesterol oxidation primarily occurs via non-enzymatic and enzymatic pathways. Non-enzymatic cholesterol oxidation includes free radical and singlet oxygen oxidations, whereas enzymatic cholesterol oxidation involves 25-hydroxylase (CH25H) and cholesterol oxidase (COD), which play critical roles [120]. CH25H catalyzes the conversion of cholesterol into 25-hydroxycholesterol (25-HC), a key oxysterol that functions as an endogenous agonist of liver X receptor alpha, thereby modulating cholesterol balance. Additionally, 25-HC serves as an inhibitory regulator, reducing cholesterol biosynthesis [121]. Meanwhile, COD catalyzes the oxidation of cholesterol to cholestenone and H_2_O_2_. Cholestenone exhibits notable antibacterial properties, whereas H_2_O_2_ production elevates ROS levels, disrupts lipid raft integrity, and induces ferroptosis. Moreover, H_2_O_2_ can function as an oxidant to generate O_2_, which inspired the development of molybdenum oxide nanodots (MONDs) as cholesterol-depleting agents, a widely recognized strategy for cholesterol metabolism regulation [94, 122–124]. Furthermore, Kobayashi et al. [124] elucidated the detailed mechanism of COD-mediated cholesterol oxidation. Initially, COD catalyzes the oxidation of the 3β-hydroxy group of cholesterol into a ketone, concurrently reducing flavin adenine dinucleotide to flavine adenine dinucleotide and H^+^. The oxidized intermediate then undergoes double-bond isomerization, yielding cholestenone. Simultaneously, the reduced flavine adenine dinucleotide and H^+^ react with molecular O_2_, producing H_2_O_2_. Notably, COD effectively mediates cholesterol oxidation within lipid bilayers and liposomal structures, leading to substantial structural alterations in lipid membranes.

### Lipid metabolic reprogramming

Lipid metabolic reprogramming is defined as the adaptive adjustment of lipid uptake, synthesis, and oxidation pathways by cells or organisms under disease conditions or altered energy demands, enabling them to cope with environmental changes or specific requirements [22]. Unlike normal lipid metabolism, this process represents a metabolic aberration that is characterized by alterations in key enzyme activity, regulatory factor expression, and metabolic flux distribution, while sharing core pathways with normal lipid metabolism. This reflects the inherent plasticity of the metabolic system, which can also act as a driver of disease progression (Fig. 2). For instance, immune cell metabolic reprogramming has been shown to support tissue repair [125]. In contrast, lipid metabolic reprogramming is widely recognized as a hallmark of malignancy and a driver of cancer progression [126]. Due to uncontrolled proliferation, tumor cells have an exceptionally high demand for oxygen and nutrients, far exceeding that of normal cells. Therefore, tumor cells reprogram their lipid metabolism to sustain survival and growth and adapt to these energetic and synthetic demands [100]. Accordingly, targeting the double-edged nature of lipid metabolic reprogramming has emerged as a major focus of current research.

In cancer, lipid metabolic reprogramming within the tumor microenvironment (TME) results in the dysregulated activation of lipid metabolic pathways [127]. Tumor cells exhibit high expression of lipid transport proteins, including CD36, FABPs, and FATPs [100]. CD36 overexpression enhances FAs uptake and intracellular lipid deposition in tumor cells, accordingly fueling tumor progression [128]; FABP5 overexpression drives epithelial-mesenchymal transition (EMT) and promotes lymphatic metastasis through FAs metabolic reprogramming, thereby facilitating tumor immune evasion [129]; and FATP2 functions as a LCFA transporter, facilitating intracellular lipid influx, particularly of arachidonic acid, the precursor of prostaglandin E2, which plays a key role in tumor-associated inflammation [130]. Moreover, within the framework of lipid metabolic reprogramming, tumor cells exhibit aberrant overexpression of key lipid biosynthetic enzymes, including ACLY and FASN. This metabolic adaptation ensures a sustained supply of metabolic precursors, which are critical for tumor proliferation and survival [100]. ACLY physically associates with β-catenin protein, facilitating its nuclear localization and enhancing its transcriptional activity. This interaction drives the transcriptional activation of downstream target genes, ultimately inducing EMT and promoting tumor cell invasion and metastasis [131]. Meanwhile, tumor cells can increase FAO levels by upregulating CPT1 expression, and high levels of FAO can promote EMT [100, 132, 133] and foster M2 macrophage polarization, which subsequently leads to the secretion of pro-inflammatory cytokines such as IL-1β, thereby augmenting tumor cell motility and proliferative capacity [134]. Tumor cells promote lipid storage to maintain an energy reservoir essential for growth. This process is driven by PPAR activation, which induces the expression of lipid storage-associated genes, including DGAT and ACAT, thereby facilitating LDs biogenesis [100]. Conversely, tumor cells upregulate lipid catabolism to fuel bioenergetic needs and to influence immune responses. Tumor cells undergo metabolic adaptation due to their rapid proliferation and frequent glucose deprivation, which leads to the phosphorylation and acetylation of choline kinase alpha 2. These modifications subsequently phosphorylate PLIN2/3, facilitating LD-ATGL interactions and promoting autophagosome-mediated LDs degradation, thereby enhancing lipolysis [135].

Notably, beyond the lipid metabolism-related proteins mentioned above that directly contribute to lipid metabolic reprogramming, oncogenes and tumor suppressor genes also play a crucial role in lipid metabolic reprogramming by modulating key lipid metabolism enzymes. For instance, myelocytomatosis virus oncogene cellular homolog (MYC), a nuclear transcription factor and proto-oncogene, orchestrates cellular processes through diverse regulatory mechanisms. Specifically, MYC governs the expression of key lipid metabolic enzymes, including ACC, FASN, and ACLY. Additionally, MYC cooperates with SREBP to activate HMGCR, driving cholesterol metabolic reprogramming to fuel tumor growth [100]. Similarly, Kirsten rat sarcoma viral oncogene homolog (KRAS), a key proto-oncogene encoding a small GTPase, is integral to metabolic reprogramming. KRAS modulates glutamine metabolism by suppressing glutamate dehydrogenase 1, enhancing pyruvate production, and increasing the availability of reducing equivalents like NADPH, thereby supplying crucial precursors for cholesterol and FAs biosynthesis [136].

Lipid metabolic reprogramming also extends beyond tumor cells, affecting various components of the TME, including immune and stromal cells. Sun et al. [137] revealed that dendritic cells (DCs) undergo lipid metabolic reprogramming, leading to excessive lipid accumulation. This process includes upregulated scavenger receptor A expression, activation of FASN, and increased LDL uptake, ultimately impairing DC function. Similarly, macrophages exhibit lipid metabolic reprogramming. M2 macrophages demonstrate enhanced lipid uptake, likely due to increased expression of lipid transporters such as CD36, which supplies energy and metabolic precursors, promoting M2 macrophage polarization. Moreover, M2 macrophages exhibit heightened FAO activity, potentially mediated by PPARδ signaling, where elevated FAO acts as a metabolic driver for M2 polarization [138]. Furthermore, Gong et al. [139] identified a metabolic shift in cancer-associated fibroblasts, characterized by increased FASN expression and a concurrent reduction in FAs degradation enzyme levels. Enzymes involved in TG and phospholipid biosynthesis are also upregulated in tumor cells, thereby reinforcing the role of lipid metabolic alterations in tumor progression.

## Lipid metabolism-related diseases

**Fig. 3:**
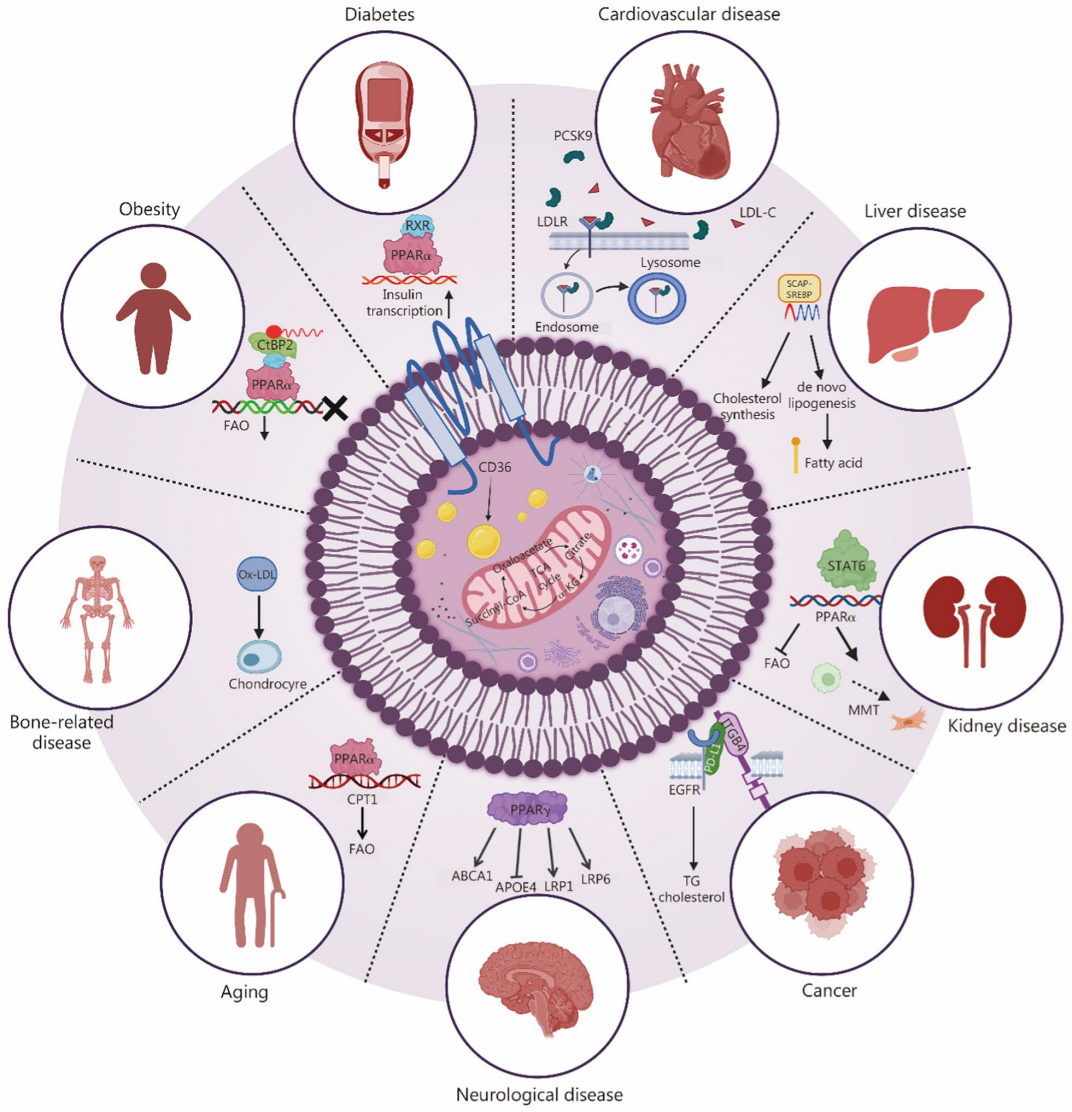
Major diseases where lipid metabolism acts as a primary or secondary driver and their lipid metabolism-associated regulatory mechanisms. Copyright Biorender.com. This image is created with BioRender. LDLR low-density lipoprotein receptor, LDL-C Low-density lipoprotein-cholesterol, FAO fatty acid β-oxidation, PCSK9 proprotein convertase subtilisin/kexin type 9, RXR retinoid X receptor, PPARα peroxisome proliferator-activated receptor α, CtBP2 C-terminal binding protein 2, OX oxidized, STAT6 signal transducer and activator of transcription 6, CPT1 carnitine palmitoyl transferase 1, ABCA1 ATP-binding cassette transporter A1, APOE4 apolipoprotein E4, LRP LDL receptor-related protein 1, PPARγ peroxisome proliferator-activated receptor γ, TG triglyceride, EGFR epidermal growth factor receptor, PD-L1 programmed cell death ligand 1, MMT mitochondrial membrane transition, SCAP SREBP cleavage-activating protein, SREBP sterol regulatory element-binding protein, CD36 cluster of differentiation 36, CoA coenzyme A, TCA tricarboxylic acid, α-KG α-ketoglutarate

Lipid metabolism plays a critical role in maintaining human health by regulating systemic energy homeostasis and modulating immune and neurological functions. Moreover, dysregulated lipid metabolism is intricately linked to the onset and progression of numerous diseases. Notably, lipid metabolism contributes to disease onset and undergoes progressive dysregulation as the disease advances, establishing a bidirectional and mutually reinforcing feedback loop. Therefore, an in-depth exploration of lipid metabolism regulation and its involvement in disease progression is crucial for advancing our understanding of disease pathophysiology and refining therapeutic strategies. This section provides a systematic analysis of the pathogenesis of diseases in which lipid metabolism acts as either a primary or secondary factor. We focus on the role of widely reported targets in various lipid metabolism segments in regulating the development of the disease, which may be the cornerstone and direction for the design of multifunctional drug delivery systems. Finally, we summarize the existing drug therapeutic strategies targeting the regulation of lipid metabolism and their advantages and disadvantages. In general, the existing drugs have limitations in terms of target specificity, safety, and adherence, and therefore, require further in-depth exploration of precise lipid metabolism interventions (Fig. 3).

### Diseases primarily driven by lipid metabolism

Diseases primarily driven by lipid metabolism encompass: 1) obesity, 2) diabetes, 3) CVDs, and 4) liver diseases. These conditions are strongly linked to metabolic dysregulation and energy equilibrium disruption, positioning them as central topics in metabolic research. Additionally, these diseases exhibit mutual interplay and reciprocal exacerbation. For instance, a strong positive correlation exists between obesity and diabetes, while both conditions substantially heighten the risk of CVD and liver pathology [140].

#### Obesity

Obesity is a multifactorial chronic metabolic disease, primarily driven by excessive nutrient intake and aberrant fat deposition. Patients with obesity exhibit increased lipid synthesis alongside compromised lipid catabolism. Notably, phosphatidylcholine, lysophosphatidylcholine, and HDL-C levels are often reduced in obesity, whereas TG, sphingomyelin (SM), and TC levels tend to be elevated. These alterations emphasize dysregulated lipid metabolism as a fundamental pathological feature of obesity [141–144]. To date, 4 key lipid metabolism regulators have been extensively investigated in obesity research: PPAR, CD36, serine active site-containing 1 (SERAC1), and adiponectin (ADPN). We further elucidate their mechanistic involvement in obesity pathogenesis and their therapeutic potential.

PPARs are a family of ligand-activated nuclear receptors that play pivotal roles in lipid metabolism and in the pathogenesis and progression of obesity [145]. The Saito group [146] employed high-fat diet-induced obese mice and genetically obese ob/ob mice as experimental models and found that malonyl-CoA levels were elevated in obese tissues, where malonyl-CoA binds to the Rossmann fold domain of C-terminal binding protein 2 (CtBP2), facilitating the interaction between CtBP2 and PPARα. This interaction suppresses PPARα activity, leading to the downregulation of FAO-related genes and subsequent lipid accumulation. Subsequently, Foretz et al. [147] discovered that small-molecule compounds capable of inhibiting malonyl-CoA synthesis, such as metformin, can disrupt the formation of the CtBP2-PPARα complex, thereby enhancing FAO and improving lipid metabolism. These findings suggest that the CtBP2-PPARα signaling pathway represents a promising therapeutic target for obesity and other lipid metabolism disorders.

CD36 functions as a key regulatory protein in lipid metabolism, and its expression is transcriptionally controlled by signal transducer and activator of transcription (STAT) factors. In particular, STAT3 directly binds to the CD36 promoter to enhance its transcription, thereby promoting adipocyte differentiation and lipid accumulation, which in turn contributes to lipid metabolic dysregulation and obesity pathogenesis [148, 149]. Notably, Su et al. [150] used in vivo and in vitro models and demonstrated that apigenin inhibits STAT3 phosphorylation, thereby disrupting the STAT3/CD36 signaling axis, reducing lipid accumulation, and preventing visceral fat deposition. Furthermore, apigenin downregulates PPARγ expression, inhibiting adipocyte differentiation, highlighting its potential as a therapeutic candidate for visceral obesity.

SERAC1 is present at ER-mitochondria contact sites, where it catalyzes phosphatidylglycerol remodeling, a crucial step in the biosynthesis of bis(monoacylglycerol)phosphate (BMP). Inadequate BMP levels disrupt cholesterol trafficking, leading to free cholesterol accumulation in endosomes. Thus, SERAC1 is essential for maintaining cholesterol balance and lipid metabolism [151, 152]. Du et al. [152] generated systemic SERAC1 knockout mice and subjected them to a high-fat diet intervention, revealing that SERAC1 deficiency elevates hepatic cholesterol while suppressing systemic cholesterol synthesis, leading to a metabolic shift favoring cholesterol production over FA synthesis. This dysregulation alters the balance between cholesterol and lipid metabolic pathways, ultimately affecting lipid accumulation and obesity progression.

ADPN, a fat-derived adipokine, plays an important role in metabolic disorders, particularly obesity. Low ADPN levels are correlated with obesity, hypertension, and diabetes [153], whereas elevated ADPN levels enhance glucose uptake and FAO via AMPK activation, improving insulin sensitivity. Moreover, ADPN directly stimulates lipolysis and mitigates fat accumulation, contributing to obesity reduction [154]. Accordingly, recent pharmacological advancements have identified thiazolidinediones such as rosiglitazone as PPARγ activators that upregulate ADPN synthesis [155, 156]. Furthermore, natural compounds like isoastragaloside-$\text{I}\!\text{I}\!$ and astragaloside $\text{I}\!\text{I}\!$ have been shown to increase ADPN secretion, offering alternative therapeutic strategies for obesity management [156].

Currently, the development of lipid metabolism-targeting anti-obesity therapies remains in its nascent phase. For instance, berberine modulates metabolism and inflammation-related genes through PPARδ activation, contributing to improved metabolic balance [157]. Similarly, apigenin exerts anti-visceral obesity effects by inhibiting the STAT3/CD36 signaling axis and suppressing lipid accumulation [150]. Additionally, orlistat, an FDA-approved anti-obesity drug, reduces dietary fat absorption by inhibiting pancreatic and gastric lipases, leading to a 30% decrease in lipid intake. However, orlistat is associated with gastrointestinal disturbances and potential interactions with drug absorption and metabolism [158–160]. Targeting lipid metabolism holds immense potential for treating obesity, but requires systematic, multi-targeted, and precision-based strategies.

#### Diabetes

Diabetes mellitus is a metabolic disorder characterized by insufficient insulin production or impaired insulin responsiveness, leading to abnormally high blood glucose levels. This disorder is closely associated with lipid metabolism dysregulation and contributes to the development of various complications [161]. Patients with diabetes typically present with elevated plasma TG, FFA, and LDL-C, alongside reduced HDL-C [162]. Additionally, distinct lipid metabolic signatures are observed across diabetic complications: upregulation of FATP, FABP, CD36, and SREBP, together with downregulation of ATP-binding cassette transporter A1 (ABCA1) promotes renal lipid accumulation and injury in diabetic kidney disease; increased LDL and reduced ABCA1 impair cholesterol efflux in diabetic retinopathy; and sphingolipid levels are elevated despite unaltered LDL in diabetic peripheral neuropathy [163–165]. These findings emphasize the complex and interconnected regulatory network governing diabetes pathogenesis, further highlighting the strong association between obesity and diabetes, as their pathophysiological mechanisms and therapeutic strategies significantly overlap [166]. Therefore, elucidating the regulatory roles of PPARs and ADPN in diabetes and its complications warrants further investigation.

Notably, PPARα and PPARδ expression levels are markedly reduced in patients with diabetes [167]. Moreover, Holm et al. [168, 169] reported that type 1 patients with diabetes mellitus exhibit a significant reduction in islet sulfatide levels compared to healthy individuals, which may be attributed to PPARα deficiency. This deficiency leads to cerebroside sulfotransferase inactivation, impaired sulfatide synthesis, and aggravated β-cell damage. Furthermore, PPARβ/δ promotes mitochondrial β-oxidation, reducing FFA accumulation in β-cells, alleviating lipotoxicity, and enhancing lipid metabolic balance by reducing SFA accumulation while increasing MUFA levels [169]. Under normal conditions, PPARγ downregulates FFA levels, thereby improving insulin sensitivity and promoting the expression of phosphatase and tensin homolog deleted on chromosome 10 (PTEN). In contrast, in the case of PPARγ deficiency or inhibition, PTEN expression is suppressed, leading to the activation of the Akt/focal adhesion kinase signaling pathway. This subsequently induces EMT in renal tubular epithelial cells, thereby exacerbating renal fibrosis and promoting diabetic kidney disease progression [170, 171].

ADPN is recognized as an independent predictor of diabetes and primarily regulates lipid metabolism via its receptors AdipoR1 and AdipoR2. ADPN functions by enhancing FAO and ceramidase activity, reducing intracellular ceramide levels, and increasing sphingosine-1-phosphate concentrations. These effects collectively contribute to the mitigation of insulin resistance and prevention of β-cell apoptosis [172, 173]. Kim et al. [173, 174] and Banerjee et al. [175] suggested that serum ADPN levels are significantly reduced in patients with prediabetes and diabetes, with ADPN levels being inversely correlated with insulin resistance, suggesting that a decline in ADPN contributes to worsening insulin resistance. Furthermore, Deng et al. [175] discovered that ADPN deficiency or dysregulation inhibits AMPK activity, leading to decreased FAO and impaired glucose balance. This reduces insulin sensitivity, promotes ceramide accumulation, and increases β-cell apoptosis, ultimately exacerbating insulin resistance and contributing to the development of diabetes and diabetic retinopathy.

As a result, the regulation of lipid metabolism in diabetes has emerged as a crucial therapeutic strategy, with several lipid-targeting drugs already in clinical use. For instance, fenofibrate activates PPARα, thereby promoting FAO, improving lipid metabolism, and reducing insulin resistance. Additionally, fenofibrate is capable of leading to a reduction in renal tubular lipotoxicity, suppression of inflammation and apoptosis, decreased proteinuria, and delayed progression of diabetic kidney disease [176, 177]. However, fenofibrate exhibits only a modest hypoglycemic effect, as its primary mechanism of action involves modulating lipid metabolism and mitigating inflammation, thereby indirectly enhancing glycemic control rather than directly influencing insulin secretion or sensitivity. Furthermore, sustained therapeutic benefits necessitate prolonged administration [176]. Shifting from “treating symptoms to lower blood sugar” to “targeting root causes to regulate metabolism” holds promise for more fundamentally improving the course of diabetes.

#### CVD

CVDs are typically caused by dyslipidemia, increased blood viscosity, and atherosclerosis (AS), often accompanied by elevated TG and TC levels. Among these factors, dysregulation of LDL-C and HDL-C levels is a fundamental driver of CVD pathogenesis and progression. To further elucidate the complicated relationship between lipid metabolism and cardiovascular pathology, this section examines the regulatory functions of PPARs, PCSK9, and cholesterol ester transfer protein (CETP)-three important lipid metabolism regulators implicated in CVD onset and progression.

In addition to their roles in obesity and diabetes, PPARs play a necessary role in cardiovascular health. PPARα facilitates FAO, effectively lowering circulating FFA and TG concentrations, thereby reducing lipid deposition in vascular walls and mitigating atherosclerotic risk [178]. Meanwhile, PPARδ plays a dual role in lipid metabolism, as it not only promotes FAO but also regulates lipid storage, thereby maintaining lipid balance and reducing CVD susceptibility [179]. Over and above that, PPARγ facilitates adipocyte differentiation, augmenting lipid storage and contributing to atheroprotective mechanisms, which may influence the progression of AS [180].

PCSK9 is a key regulator of lipid metabolism, capable of binding to LDLR and facilitating its degradation, thereby reducing LDLR levels. Additionally, PCSK9 promotes foam cell formation, wherein these cells engulf cholesterol and contribute to the development of AS plaques [181, 182]. While gain-of-function mutations in PCSK9 lead to familial hypercholesterolemia or an increased risk of AS, loss-of-function mutations exhibit a protective effect against AS [182, 183].

CETP primarily regulates cholesterol transport among HDL, LDL, and VLDL. Under normal physiological conditions, CETP maintains lipid equilibrium; however, its overexpression can lead to elevated LDL-C levels, increasing the likelihood of arterial plaque formation and heightening the risk of CVDs [184–186]. On top of that, Dunca et al. [187] observed an interesting phenomenon that CETP mutations exhibit distinct effects in different populations of European cohorts, CETP variants influence both HDL-C and LDL-C levels, whereas in East Asian populations, these mutations predominantly affect HDL-C, with minimal impact on LDL-C concentrations.

A growing body of evidence supports the role of lipid-modulating agents in CVD prevention and treatment. Clinical studies indicate that fibrate-class agents, including fenofibrate, mitigate CVD risk and attenuate AS plaque progression through PPARα activation [188]. Moreover, PCSK9 inhibitors such as alirocumab and evolocumab promote LDL-C clearance by blocking PCSK9 function, thereby ameliorating CVDs such as AS and angina [189, 190]. Nevertheless, concurrent administration with statins may disrupt statin metabolism, increasing plasma drug levels and predisposing patients to adverse reactions. Overall, lowering LDL-C remains the most effective cardiovascular risk intervention demonstrated to date. The future lies in precisely targeting residual cholesterol, systemic anti-inflammation, and achieving ultra-long-acting management through revolutionary technologies.

#### Liver disease

The liver is the primary site of lipid synthesis and metabolism, and thus, hepatic disorders are frequently associated with lipid metabolic dysregulation. For example, metabolic dysfunction‑associated fatty liver disease (MAFLD) and nonalcoholic fatty liver disease (NAFLD) are predominantly characterized by hepatic lipid accumulation, leading to elevated levels of TG, FFA, and cholesterol [191–193]. Conversely, cirrhosis is marked by excessive lipid catabolism, manifesting as increased FFA concentrations and decreased TG levels. Meanwhile, in liver failure, impaired lipid biosynthesis results in reduced TG and cholesterol levels, alongside elevated FFA concentrations, thereby aggravating metabolic dysfunction, inflammatory responses, and hepatocellular injury [194–197]. Although the patterns of lipid alterations vary among hepatic diseases, they collectively emphasize the important role of lipid metabolic dysregulation in liver disease pathogenesis and progression.

SREBPs are a family of transcription factors that are essential for lipid metabolism and equilibrium, regulating the expression of genes involved in lipid biosynthesis and uptake to control cholesterol and FA synthesis and absorption. Their activation is modulated by ER feedback mechanisms and external signaling pathways, including transforming growth factor-β-activated kinase 1 (TAK1) and PLIN2 [10, 198, 199]. Under physiological conditions, TAK1 restrains excessive SREBP activation, thereby preserving hepatic lipid homeostasis. Conversely, TAK1 deficiency or dysregulation leads to SREBP hyperactivation, resulting in increased TG and cholesterol accumulation, excessive lipid synthesis, and heightened susceptibility to hepatic disorders such as fatty liver degeneration and liver fibrosis [199]. In contrast, PLIN2 deficiency suppresses SREBP activation, downregulating key lipid metabolic genes such as FASN and ACC, reducing hepatic accumulation of neutral lipids (TG and cholesterol), modulating membrane lipid fluidity, alleviating ER stress, and contributing to NAFLD prevention and mitigation [198].

SREBP cleavage-activating protein (SCAP) is an important regulator of SREBP activation, which is essential for lipid homeostasis. SCAP forms a complex with insulin-induced gene (INSIG) proteins and modulates SREBP transport from the ER to the Golgi apparatus for activation. Under elevated intracellular cholesterol conditions, SCAP interacts with INSIG, sequestering the SCAP/SREBP complex within the ER, thereby suppressing lipid biosynthesis. Conversely, cholesterol depletion induces a structural rearrangement in SCAP, facilitating SREBP release and subsequent activation. Once SREBP reaches the Golgi apparatus, proteolytic cleavage of lipid peroxidation (LPO) enables its nuclear translocation, where it drives the expression of lipid metabolic genes [200, 201]. In contrast, aberrant SCAP/SREBP activation exacerbates hepatic TG accumulation, triggering ER stress and lipotoxicity, ultimately contributing to hepatitis and liver fibrosis [201, 202]. Notably, histone deacetylase 3 (HDAC3) and the SCAP/SREBP axis independently regulate hepatic lipid metabolism. While HDAC3 primarily represses lipid synthesis and promotes FAO, SCAP/SREBP predominantly facilitates lipid and TG biosynthesis. These 2 pathways function in a compensatory manner, ensuring hepatic lipid balance under normal conditions. However, simultaneous impairment of both pathways induces oxidative stress and inflammation, culminating in severe hepatic injury [203].

Therefore, targeting lipid metabolism has emerged as a promising therapeutic strategy for liver diseases, encompassing approaches such as modulating lipid accumulation and activating PPARs. For instance, antrodan alleviates NAFLD by modulating the AMPK-SREBP-1c-PPARγ pathway, while xanthohumol ameliorates hepatic steatosis by inhibiting the SCAP/SREBP complex [204]. Moreover, several pharmacological agents targeting lipid metabolism have been introduced for liver disease therapy. For example, fenofibrate exerts hepatoprotective effects by activating PPARα, thereby suppressing c-Jun N-terminal kinase (JNK) signaling and mitigating hepatic injury. Moreover, it downregulates bile acid synthesis genes while promoting bile acid excretion, thereby preventing bile acid accumulation and associated hepatic damage [205]. However, the SREBP signaling pathway is ubiquitously expressed across various tissues and is integral to lipid metabolism, cholesterol equilibrium, and insulin signaling. Therefore, systemic inhibition of SREBP may elicit unintended off-target effects in non-hepatic tissues, necessitating precise therapeutic modulation. Many metabolic pathways are widely distributed throughout the body, making liver-specific or highly selective drugs crucial for reducing systemic side effects. Furthermore, innovations in diagnostic and monitoring technologies will significantly accelerate clinical trials and long-term patient management, such as through blood biomarkers and novel imaging techniques for liver fat and liver stiffness.

### Diseases secondarily influenced by lipid metabolism

Lipid metabolism dysregulation is implicated as a secondary contributor in several diseases, including: 1) kidney diseases, 2) cancer, 3) neurological disorders, 4) aging, and 5) bone-related diseases. Although lipid metabolism abnormalities are not the primary cause of these conditions, they play a significant role in modulating disease progression through diverse regulatory mechanisms, which can influence pathological processes, making lipid metabolism a promising therapeutic target.

#### Kidney disease

Lipid metabolism plays an indispensable role in maintaining kidney function, supplying energy and protective effects for normal renal physiology. Subsequently, disruptions in lipid metabolism contribute to the pathogenesis of various kidney diseases, with distinct lipid metabolic alterations characterizing different renal disorders. For instance, TC, TG, and LDL levels are positively correlated with proteinuria in tubulointerstitial nephropathy, whereas elevated TG levels and reduced LDL levels are observed in chronic kidney disease [206–209].

PPARs are one of the major contributors to lipid metabolism. Among the PPAR isoforms, PPARα plays a particularly prominent role in renal disease, exerting complex and multifaceted regulatory effects. Cheng et al. [210] revealed that PPARα activation enhances Wnt signaling, thereby amplifying inflammatory responses and suppressing renal tubular epithelial cell proliferation, ultimately driving renal fibrosis. In contrast, PPARα downregulation disrupts FAO equilibrium by reducing FABP1 expression, which increases oxidative stress, promotes ferroptosis, and accelerates the progression of immunoglobulin A nephropathy [211]. Furthermore, PPARα interacts with STAT6, leading to STAT6 inhibition and FAO suppression. This promotes lipid accumulation in renal tubular epithelial cells and macrophages, which triggers EMT in tubular cells, and reprograms macrophage metabolism, respectively, thereby promoting their transition from anti-inflammatory M2 macrophages to pro-fibrotic myofibroblasts. Collectively, these changes culminate in renal fibrosis [212, 213].

Thus, PPAR-targeted modulation of lipid metabolism has emerged as a promising therapeutic approach for renal disorders. PPARα agonists, such as fenofibrate and bezafibrate, effectively lower lipid levels and mitigate renal injury by reducing glycoproteinuria and glomerular damage [214]. Similarly, other PPARγ agonists improve insulin sensitivity, suppress inflammation, and attenuate renal fibrosis [215]. However, PPARγ agonists are associated with adverse effects such as fluid retention, peripheral edema, and increased blood volume, which limit their widespread clinical application. Kidney diseases with different etiologies, such as diabetic nephropathy, focal segmental glomerulosclerosis, and hypertensive nephropathy, may exhibit distinct patterns of lipid metabolism disorders, necessitating individualized treatment strategies.

#### Cancer

Cancer is characterized by high invasiveness and metastatic potential, making it one of the leading causes of mortality worldwide. Unlike normal cells, cancer cells evade contact inhibition, allowing for uncontrolled proliferation, infiltration into surrounding tissues, metastasis, and resistance to apoptosis. Cancer cells dynamically reshape their lipid metabolism via lipid metabolic reprogramming to support macromolecular biosynthesis, meet energy demands, enhance signal transduction, and evade programmed cell death. Expanding on previous discussions of lipid metabolic reprogramming, this section delves into the roles of SREBP, Liver X receptors (LXR), PPAR, and MGLL in tumor-associated lipid metabolism, examines oncogene-driven lipid metabolic regulation across various cancers, and explores lipid metabolic reprogramming in other components of the TME.

SREBP is frequently upregulated in various cancers, potentially through tumor cell-expressed Programmed Cell Death Ligand 1, which activates the EGFR/integrin subunit β4/SREBP1c signaling axis, thereby driving lipid metabolic reprogramming and supporting tumor progression [22, 216]. Additionally, LXR and PPAR have also been extensively investigated in the context of lipid metabolic reprogramming in cancer [217]. For instance, LXR modulates lipid metabolism in colorectal cancer (CRC) by suppressing cholesterol absorption, enhancing cholesterol excretion, and inhibiting cholesterol biosynthesis. In addition, LXR regulates the balance between SFAs and MUFAs, thereby influencing tumor cell proliferation [218]. Similarly, PPARα plays a crucial role in cancer lipid metabolism reprogramming by promoting FAO. Specifically, PPARα induces FAO-related gene expression, facilitating FFA breakdown to generate energy for cancer cells. Furthermore, PPARα activation enhances the expression of angiopoietin-like protein 4, a key regulator of cellular motility, thereby promoting tumor invasion and metastasis [219]. MGLL exhibits dual roles in cancer, acting as either a tumor suppressor or an oncogenic factor, depending on the cancer type. MGLL is typically downregulated in colorectal and liver cancers, functioning as a tumor suppressor. Conversely, MGLL expression is upregulated in breast and prostate cancers, facilitating tumor progression [220–222]. Additionally, LDs serve as reservoirs of FAs, which, upon hydrolysis, fuel myeloid-derived suppressor cells (MDSCs) and tumor-associated macrophages (TAMs). This metabolic support enhances the survival and immunosuppressive functions of these immune cells, thereby promoting tumor immune evasion [223].

In hepatocellular carcinoma (HCC), Yin Yang 1 serves as a key suppressor of FAO. Particularly, it inhibits the activity of peroxisome proliferator-activated receptor gamma coactivator-1β, leading to the downregulation of medium-chain acyl-CoA dehydrogenase (MCAD) and long-chain acyl-CoA dehydrogenase (LCAD). Accordingly, this suppression reduces mitochondrial FAO, facilitating lipid accumulation and promoting tumor growth [224]. Similarly, Morales et al. [225] demonstrated that loss of ephrin transmembrane receptor subclass B2 (EPHB2) in prostate cancer expression leads to LDs accumulation, which enhances tumor cell proliferation, migration, and immune evasion [226]. Accordingly, targeting lipid metabolism regulators such as DGAT1 has emerged as a promising therapeutic strategy for tumors deficient in EPHB2. Moreover, FAO is highly upregulated in MYC-overexpressing triple-negative breast cancer (TNBC), and treatment with FAO inhibitors such as etomoxir effectively impairs tumor growth, suggesting that FAO inhibition represents a potential therapeutic strategy for TNBC [227]. In addition, the HMGCR degradation protein 1 expression is markedly reduced in TNBC cells, while CPT2 activity is significantly elevated. The synergistic effect of these 2 factors contributes to FAO upregulation in TNBC, further supporting its role as a potential therapeutic target [228].

Lipid metabolic reprogramming within the TME also significantly influences tumor progression and immune evasion. Excessive accumulation of LCFAs impairs mitochondrial function in CD8^+^ T cells, thereby reducing their antitumor activity [229]. Furthermore, increased FA synthesis promotes regulatory T cell (Treg) differentiation, enhancing their immunosuppressive phenotype and further dampening the antitumor immune response [230]. Moreover, lipid metabolism is intricately linked with glucose and amino acid metabolism, collectively shaping the metabolic landscape of the TME and influencing tumor progression and immune modulation [229].

Currently, several cancer treatment strategies targeting lipid metabolism are under active investigation. One promising approach involves targeting lipid metabolic enzymes such as ACSL and utilizing ferroptosis inducers like RSL3. These strategies promote LPO in tumor cells, ultimately triggering ferroptosis regulated form of cell death driven by oxidative damage to lipid membranes [231]. Meanwhile, targeting FASN and sphingosine kinases has emerged as a promising therapeutic avenue in hematologic malignancies, which has demonstrated potential in disrupting lipid metabolism to impair cancer cell survival [232]. Furthermore, a recent study suggests that pharmacological inducers or RNA interference (RNAi) targeting cytosolic phospholipase A2 α can modulate T-cell lipid metabolism, thereby reducing LDs accumulation, mitigating T-cell exhaustion, and ultimately enhancing antitumor immunity. This strategy represents a novel direction for cancer immunotherapy [233]. Despite these advances, no clinically approved anticancer drug directly targeting lipid metabolism is currently available. Therefore, further research is necessary to refine lipid-targeting strategies, optimize therapeutic efficacy, and establish clinical safety profiles through rigorous validation.

#### Neurological diseases

Neurological disorders are frequently associated with profound disturbances in lipid metabolism. In Alzheimer’s disease (AD), monoacylglycerol and DAG levels are elevated, whereas glycerophospholipid and PUFA levels are reduced, which compromises neuroprotective capacity. In Parkinson’s disease (PD), FAO is impaired, characterized by reduced fatty acyl groups and triglycerides alongside elevated SM and CE [234–236]. Similar lipid-neurological associations are observed in other disorders: multiple sclerosis features reduced brain sphingolipids leading to myelin loss and neuronal injury; stroke-induced hypoxia triggers lipid peroxidation and deposition; and excess brain cholesterol lowers neuronal excitation thresholds, precipitating epilepsy [237]. Within these pathological contexts, PPAR and PCSK9, as pivotal regulators of lipid metabolism, have emerged as central players in the onset and progression of neurological diseases.

PPARs, well-known for their roles in obesity, diabetes, CVD, and kidney disorders, also regulate critical processes in the nervous system, particularly in AD pathogenesis. The abnormal accumulation of amyloid β-protein is a major contributor to AD. PPARγ enhances ABCA1 expression, facilitating cholesterol efflux and reducing intracellular cholesterol accumulation. Additionally, PPARγ upregulates low-density lipoprotein receptor-related protein 1, promoting amyloid β-protein clearance, thereby mitigating AD progression [238]. Given that lipids constitute a major component of the nervous system, PCSK9 plays a key role in maintaining lipid balance by interacting with LDL receptor family proteins and CD36 [239]. Jaafar et al. [240] first demonstrated the critical role of PCSK9 in maintaining peripheral nerve metabolic homeostasis, which extends beyond its classical function in LDL-C regulation. CD36 expression in the sciatic nerve is upregulated in the absence of PCSK9, accompanied by increased cholesterol and TG levels and LDs accumulation, culminating in peripheral nerve lipid overload, mitochondrial dysfunction, and the onset of sensory neuropathy.

Subsequently, the importance of lipid metabolism in neurological disorders has garnered increasing attention. A recent study suggests that LXR agonists can enhance ABCA1 expression, thereby facilitating lipid clearance, preventing lipid accumulation, and alleviating forebrain atrophy, ultimately improving AD outcomes [241]. However, LXR receptors are widely distributed across multiple tissues and cell types, particularly in immune and metabolic regulatory pathways. Therefore, their activation may lead to unintended immunosuppressive effects or metabolic imbalances, necessitating careful evaluation in clinical applications.

#### Aging

Aging can be categorized into cellular aging and organismal aging, both of which are accompanied by alterations in lipid metabolism. The lipid metabolism of senescent cells is a complex process. Long-lived species generally exhibit a lower PUFA/MUFA ratio than short-lived species, as this ratio governs membrane fluidity and oxidative vulnerability. Since PUFAs are highly susceptible to peroxidation by ROS, a lower PUFA/MUFA ratio may enhance cellular resilience against oxidative stress, which is a key driver of aging [242–244]. Additionally, brain cholesterol, primarily stored in myelin and neuronal plasma membranes, declines with age, which is associated with synaptic dysfunction and an increased risk of neurodegenerative diseases [244]. Similarly, age-associated phospholipid remodeling has been observed, where glycerophospholipid metabolism undergoes significant shifts. For instance, elevated phosphatidylglycerol and phosphatidylinositol levels contribute to membrane fluidity adjustments, potentially shielding cells from environmental stressors [245]. Beyond these lipid classes, sphingolipid metabolism also plays a crucial role in aging. For example, a decline in sphingosine kinase levels leads to sphingosine accumulation, which accelerates cellular senescence. Similarly, upregulated sphingosine synthase activity fosters sphingosine biosynthesis, further exacerbating senescence-related phenotypes [246]. Considering the important role of lipid metabolism in aging, we reviewed the regulatory influence of PPAR and SREBP in aging-related processes.

PPARα activates CPT1, enhancing FAO and promoting ROS generation, which in turn induces mitochondrial dysfunction and triggers aging-associated signaling cascades, including the p53 pathway [247]. In contrast, PPARγ deficiency or suppression has been shown to delay thymic aging and bolster immune function [248]. SREBP1 expression is upregulated during cellular senescence, leading to increased lipogenesis and membrane lipid accumulation, contributing to organelle hypertrophy, a hallmark of senescent cells. Additionally, SREBP1 overexpression induces p21 and p16 expression, thereby inhibiting the cell cycle and reinforcing cellular senescence [249]. Furthermore, SREBP2, apart from its role in lipid biosynthesis, induces the expression of senescence-associated β-galactosidase, thereby promoting hepatocyte senescence [250].

Emerging evidence emphasizes the therapeutic potential of lipid metabolism modulation in mitigating aging-related decline. For instance, fenofibrate, a PPAR agonist, has been shown to regulate lipid metabolism, enhance FAO, and reduce senescent chondrocyte accumulation in osteoarthritis, thereby attenuating inflammatory microenvironments and exerting protective effects [246]. Additionally, a combination of rifampicin, psora-4, and allantoin has been reported to upregulate MUFA levels and delay senescence via the SREBP signaling pathway [251]. Despite these promising preclinical findings, the complexity of aging as a systemic process involving multi-organ functional decline presents challenges for single-target interventions. Accordingly, although modulating lipid metabolism holds promise as an anti-aging strategy, it is unlikely to fully counteract aging-related deterioration, necessitating complementary therapeutic approaches.

#### Bone-related diseases

Bone-related diseases, such as osteoarthritis (OA) and osteoporosis (OP), are intricately associated with lipid metabolism, as abnormal lipid metabolism can exacerbate bone density loss and compromise bone quality, ultimately contributing to disease onset [252]. Wang et al. [253] performed multi-omics profiling of human tissues and biological fluids, and found that metabolic analyses identified alterations in linoleic acid and glycerophospholipid metabolism in OA, which may contribute to cartilage degeneration by disrupting membrane lipid composition and chondrocyte function. Meanwhile, in OP, elevated cholesterol levels have been shown to regulate osteoblast and osteoclast activity, potentially favoring bone resorption over bone formation, thereby disturbing bone balance [254]. Furthermore, the downregulation of FA metabolism-related genes in postmenopausal women leads to excessive lipid deposition in skeletal muscle, adipose tissue, and bone marrow, which in turn impairs FAO. This metabolic dysfunction promotes osteoclast differentiation, exacerbating bone loss and contributing to OA progression [255].

Given that SREBP is a key lipid metabolism regulator, in bone-related pathologies, the dysregulation of SREBP2 signaling has been implicated in the pathogenesis of OA. Kostopoulou et al. [256] used human primary chondrocytes and the TC28 chondrocyte line in vitro, and a mouse OA model in vivo, and demonstrated that TGF-β expression is significantly upregulated in OA chondrocytes. This, in turn, stimulates SREBP2 activity via integrin alpha V-mediated activation of the PI3K/Akt pathway, and enhances the activation of HMGCR. Hence, cholesterol biosynthesis is markedly increased in OA, leading to cholesterol accumulation within chondrocytes. This metabolic shift induces cellular stress, disrupts chondrocyte balance, and accelerates cartilage matrix degradation, ultimately exacerbating OA progression.

OA cartilage is not merely an inert, degenerative tissue but instead undergoes active yet aberrant metabolic reprogramming. Thus, SREBP2, as a central regulator of this metabolic dysregulation, represents a highly promising therapeutic target [256]. For instance, fatostatin, an SREBP activation inhibitor, effectively suppresses the activation and maturation of SREBP2. Accordingly, it inhibits osteoclast differentiation and downregulates the expression of osteoclast marker genes, thereby ameliorating osteoporosis and other bone disorders [257]. Whereas, as previously noted, SREBP plays a critical role in osteoclast function and lipid balance across multiple tissues and organs. Thus, the systemic inhibition of SREBP by fatostatin may lead to unintended disruptions in lipid metabolism in non-target tissues, necessitating more selective therapeutic strategies.

## Representative advancements and applications of nano-delivery systems focusing on the regulation of lipid metabolic processes

The complicated relationship between lipid metabolism and disease pathogenesis has been widely recognized as a biological challenge, given that each step of lipid metabolism plays a critical role in maintaining energy balance, membrane integrity, hormone biosynthesis, immune function, and cardiovascular health. Moreover, the availability of pharmacological agents capable of selectively regulating lipid metabolism with high precision remains limited due to the highly interconnected nature of lipid metabolic processes and their complex regulatory networks [113]. This emphasizes the urgent need for in-depth exploration of precisely targeted drug delivery strategies and the development of multi-pathway intervention approaches, which may offer more effective therapeutic solutions for restoring lipid balance, preventing metabolic disorders, and improving overall health outcomes.

Lipid metabolism is governed by a network of key enzymes, transport proteins, and receptors, many of which are associated with multiple physiological processes. Hence, when these targets are disrupted or pharmacologically regulated, the effects may extend beyond lipid metabolism itself, potentially triggering a cascade of secondary physiological alterations [258]. Therefore, the design of drug delivery systems targeting lipid metabolic processes must ensure that the therapeutic agent is active after administration while being able to cross the complex metabolic environment to precisely reach the desired target. Furthermore, the carrier system and its metabolic byproducts must be formulated to exhibit minimal or no toxicity to ensure optimal safety. Here, we review the latest advancements and representative applications of drug delivery platforms in different diseases targeting key lipid metabolic processes, including lipid uptake, synthesis, storage, lipolysis, oxidation, and metabolic reprogramming (Table 1) [20, 137, 222, 259–288]. NPs and liposomes remain the most widely investigated and established platforms of nanocarriers. Meanwhile, emerging biomaterials such as engineered adipocytes, extracellular vesicles secreted by natural cells, stimuli-responsive nano-micelles, composite hydrogels, and bioengineered LDs are also gaining increasing attention for their potential in precision lipid metabolism regulation. Furthermore, advances in drug delivery strategies-incorporating gene-editing technologies, biomimetic modifications, and multi-target interventions, revolutionize target specificity, drug bioavailability, immune modulation, and the ability to overcome biological barriers, ultimately improving therapeutic efficacy and safety.

**Table 1:** Summary of the multifunctional platform application in different lipid metabolism-related diseases with a specific target

Lipid metabolic process	Target	Disease	Multifunctional platform	Loaded drug	Dosage	Drug loading dose/Encapsulation efficiency	References
Lipid uptake	FABP4	Melanoma	pDox + RA@adipocytes	Doxorubicin prodrug	500 nmol/L (in vitro), 0.10 mg/kg (in vivo)	About 0.60 μg/10⁶ cells/Not mentioned	[259]
		Aging	Pd/hCeO_2_-BMS309403@platelet membrane	BMS309403 (FABP4 inhibitor)	Not mentioned	8.20 wt%/Not mentioned	[260]
	CD36	Cardiovascular disease	PA/ASePSD	Astaxanthin, PMeTPP-MBT	Not mentioned	8.90 wt% (Astaxanthin), 4.70 wt% (PMeTPP-MBT)/Not mentioned	[261]
		Obesity	ACAT2 siRNA/CS-PLGA	ACAT2 siRNA	40 μg/kg (in vivo)	Not mentioned/85.00%	[262]
	SR-B1	Ovarian cancer	HDL-like nanoparticles	Bioinspired HDL	1 μmol/L (in vitro)	-	[263]
Lipid synthesis	FASN, SCD1, SREBF1, DGAT2	Obesity	PAMAM-G3-Chol (5) nanoparticles	Cholesterol	10 mg/kg (in vivo)	-	[264]
	FASN, ACC1, SREBP1	NAFLD	GA-LpEVs-FX (Extracellular vesicles)	Fucoxanthin	10 mg/kg (in vivo)	Not mentioned/86.00%	[265]
	HMGCR	Breast cancer	Fe_3_O_4_@PCBMA	Simvastatin (HMGCR inhibitor**)**	4 mg/kg (in vivo)	15.00%/Not mentioned	[266]
	ACAT, SREBP2	Breast cancer	EALP (liposome)	Avasimibe, Phosphorbide A	4.13 mg/kg (Avasimibe in vivo), 3.66 mg/kg (Phosphorb in vivoide A)	(3.520 ± 0.004)% (Avasimibe), (3.100 ± 0.250)% (Phosphorbide A)/(87.450 ± 0.810)% (Avasimibe), not mentioned (Phosphorbide A)	[267]
	ACC1, USP22, PPARγ	Liver cancer	NTA630-NCs-RBCM-T	Cas9 RNP (USP22 inhibitor), ND630 (ACC1 inhibitor)	About 10 μg every 3 d (Cas9 RNP in vivo)/200 μg every 3 d (ND630 in vivo)	67.90% (Cas9 RNP), not mentioned (ND630)/8.50% (Cas9 RNP), not mentioned (ND630)	[20]
	SCD1	Ovarian cancer	Aur/Plu@HM (Micelles)	PluriSin1 (SCD1 inhibitor), Auranofin (ferroptosis inducer)	5 mg/kg (Auranofin in vivo), 5 mg/kg (PluriSin1 in vivo)	3.60% (Auranofin), 2.40% (PluriSin1)/Not mentioned	[268]
	PNLIP, DGAT2	Colorectal cancer	Liposome-Ma	Matairesinol	Not mentioned	9.80%/85.00%	[269]
Lipid storage and lipolysis	Lipid droplet	Breast cancer	DC@AIE-dots	MeTIND-4 (photosensitiser)	Not mentioned	Not mentioned	[270]
		Obesity	TTMN, MeTTMN	TTMN, MeTTMN	2 μmol/L (in vitro), 50 μmol/L (in vivo)	**–**	[271]
	Adipocyte	Ovarian cancer	Pyrolipid@LDs	Photosensitiser	Not mentioned	Not mentioned	[272]
	ATGL, HSL	Obesity	Rec-tNVs (Plant-derived nanovesicles)	Turmeric	40–50 mg/kg (in vivo)	1.32 × 10⁸ molecules/Particle/77.90%	[273]
	PPARγ	Obesity	MiR-130b-MV (Microvesicles)	MiR-130b	Not mentioned	Not mentioned	[274]
Lipid Oxidation	**–**	Liver fibrosis	CS-NPs/VDG, GA-NPs/SIB	Vismodegib, silybin	50 mg/kg (CS-NPs/VDG in vivo), 50 mg/kg (GA-NPs/SIB in vivo)	(1.63 ± 0.01)% (CS-NPs/VDG), (0.96 ± 0.01)% (GA-NPs/SIB)/(91.89 ± 1.14)% (CS-NPs/VDG), (98.26 ± 0.48)% (GA-NPs/SIB)	[275]
	**–**	Osteoarthritis	Ce@D&P	Deferasirox	2.50 μg/ml (both in vitro and in vivo)	**–**	[276]
	PPARα, CPT1	NAFLD and obesity	**–**	TiO_2_, Au, NaYF_4_	0.72–18.00 mg/kg (in vivo)	**–**	[277]
			CDCA-GNPs	Chenodeoxycholic acid	10, 50, 100 μmol/L (in vitro)	About 9.09%/(91.60 ± 0.30)%	[278]
	COD	Breast cancer	DOX@MOF-COD@CS	COD	5 mg/kg (in vivo)	About 16.67%/Not mentioned	[279]
		Bladder cancer	MONDs	COD	20 mg/kg (in vivo)	**–**	[280]
		Liver cancer, breast cancer	DA-COD-OD-HCS (Hydrogels)	Dextran, hemin, COD	10 mg/ml (0.5–1.0 mg in mouse, 1 mg in rat)	**–**	[281]
Lipid metabolic reprogramming	**–**	Prostate cancer	GBP@Fe_3_O_4_	Fe_3_O_4_, 1H-PFP	16 mg Fe/kg (in vivo)	11.40%/Not mentioned	[282]
	Msr1, ACC, lipogenic genes	Breast cancer	TS-PP@FU	TOFA (ACC inhibitor), STF (XBP1 inhibitor)	20 mg/kg (in vivo)	(14.70 ± 0.60)% (TOFA), (15.70 ± 2.30)% (STF)/(93.40 ± 1.30)%	[137]
	**–**	Colorectal cancer, melanoma	TOFA@PLGA@OMs-PLs	TOFA	13.30 μg/ml (in vitro), 23.28 μg/mouse	7.76%/21.29%	[283]
	PPARα, CPT1, CD36	Melanoma	aCD3/F/AN	Fenofibrate (PPARα agonist)	18 μg/mouse	Not mentioned	[284]
	MGLL, CB-2	Pancreatic cancer	Reduction-responsive RNAi	siMGLL, siCB-2	1 nmol/L (in vitro)	0.18%/80.00%	[222]
	COD	Osteoarthritis	LCF-CSBN	Licofelone (inhibitor of COD and LOX)	0.0098–2.5000 μg/ml (in vitro), 1 μg/μl (in vivo)	(4.75 ± 0.76)%/(94.30 ± 2.74)%	[285]
	PLIN2	NAFLD	FPPD	FP (ROS scavenger)	0.01 mmol/(L‧kg) (in vivo)	44.40%/Not mentioned	[286]
	SREBP, CD36, PPARα, PLIN	Wound biofilm infections	HSA-IR820@OA@ZIF-8	HSA-IR820, Oleic acid	500 μg/ml (in vitro)	Not mentioned	[287]
	CD36, CPT1	Breast cancer	iF-CuS-M/SSO@Gel (hydrogels)	iFSP1, SSO (CD36 inhibitor)	7 mg/kg iFSP1 in 10 mg/kg CuS NPs and 25 mg/kg SSO	About 60.00% (iFSP1), not mentioned (SSO)/Not mentioned	[288]

*FABP* fatty acid-binding protein, *pDox* doxorubicin prodrug, *RA* rumenic acid, *CD36* cluster of differentiation 36, *ACAT* acyl coenzyme A-cholesterol acyltransferase, *SR-B1* scavenger receptor class B type 1, *HDL* high-density lipoprotein, *FASN* fatty acid synthase, *SCD* stearoyl-CoA desaturase, *SREBF1* sterol regulatory element-binding transcription factor 1, *DGAT* diacylglycerol acyltransferase, *ACC* acetyl-CoA carboxylase, *NAFLD* nonalcoholic fatty liver disease, *HMGCR* 3-hydroxy-3-methylglutaryl (HMG)-CoA reductase, *SREBP* sterol regulatory element-binding protein, *USP22* ubiquitin specific peptidase 22, *PPARγ* peroxisome proliferator-activated receptor γ, *PNLIP* pancreatic lipase, *ATGL* adipose triglyceride lipase, *HSL* hormone-sensitive lipase, *PPARα* peroxisome proliferator-activated receptor α, *CPT1* carnitine palmitoyl transferase 1, *COD* cholesterol oxidase, *Msr1* macrophage scavenger receptor 1, *MGLL* monoglyceride lipase, *PLIN* perilipin, *NPs* nanoparticles, *SIB* silybin

### Targeted intervention in lipid uptake

The process of lipid uptake can be further subdivided into transport, recognition, binding, and translocation, which are typically regulated by specific receptors and channel proteins. These include FA uptake-related proteins such as FABPs, FATPs, and CD36, as well as cholesterol uptake-related proteins such as ACAT2, LDLR, and scavenger receptors. Dysfunctions in these lipid uptake proteins can lead to abnormal lipid accumulation or metabolic dysregulation, thereby increasing the risk of CVDs, obesity, fatty liver disease, and cancer [289, 290]. Therefore, designing appropriate carriers to deliver inhibitors targeting these molecules or other drugs capable of regulating these targets is considered an effective strategy for lipid uptake intervention.

FABP4, a member of the FABP family and an essential lipid chaperone, has been identified as a key protein involved in facilitating intracellular FA uptake and targeted localization [291]. Utilizing the FABP4-mediated lipid metabolism uptake pathway, Gu et al. [259] designed a novel doxorubicin prodrug (pDox) that responds to ROS in the TME. Additionally, they ingeniously combined pDox with an anticancer FA, rumenic acid (RA), and encapsulated both drugs within adipocytes to form pDox + RA@adipocytes. This biomimetic drug delivery system improved drug loading capacity and transformed adipocytes into tumor-specific bioresponsive drug reservoirs, allowing for precise and sustained drug release. Subsequently, computational modeling and quantitative characterization confirmed that pDox exhibited a high binding affinity for FABP4, facilitating cancer cell uptake through lipid metabolism processes and significantly enhancing drug transport efficiency. Moreover, in vivo experiments using a B16F10 mouse melanoma model demonstrated that injection of pDox + RA@adipocytes significantly delayed tumor growth, accompanied by increased infiltration of CD4^+^ and CD8^+^ T cells into the tumor tissue and a reduction in the number of Tregs. In parallel, in vitro co-culture studies using 3T3-L1 differentiated adipocytes with various cancer cell lines in a Transwell system showed that pDox + RA@adipocytes exerted enhanced cytotoxicity against cancer cells. This enhanced efficacy is attributed to the ROS-responsive linker, which improves tumor-specific drug activation, and to the engineered adipocytes serving as a “Trojan Horse”, utilizing the metabolic pathways of the cancer cells for targeted intracellular drug delivery. Notably, the tumor-induced LDs degradation process further accelerated drug release from adipocytes, allowing both chemotherapeutic agents and anticancer FAs to be released alongside FFAs, ensuring their efficient uptake by cancer cells via tumor-specific metabolic pathways. This study represents the first successful application of lipid metabolism pathways for anticancer drug delivery, and proposes a novel strategy that uses the metabolic pathway “normal adipocytes transport FAs to cancer cells for energy supply” to transport drugs into cancer cells. Subsequently, this pioneering drug delivery approach holds significant potential for expanding into other lipid metabolism-related diseases, given that lipid metabolic pathways are a major contributor to tumor chemoresistance. Li et al. [260] also reported a Pd/hCeO_2_ heterostructure with a hollow cavity and developed a Pd/hCeO_2_-BMS309403@platelet membrane (PCBP) nanocapsule system encapsulating an FABP4 inhibitor. This system was designed to counteract aging by stimulating vascular endothelial cell regeneration and suppressing FABP4 accumulation, thereby restoring glucose-lipid metabolic balance. BMS309403 is an FABP4-targeting inhibitor that regulates FA transport and metabolism, thereby mediating age-related obesity and influencing aging-related diseases [292]. The PCBP system is composed of platelet membrane-coated Pd/hCeO_2_, where the platelet surface modification confers biomimetic properties, enabling immune evasion and inflammation-specific targeting. Mechanistic investigations demonstrated that the PCBP system efficiently eliminated ROS, in turn stimulating vascular endothelial growth factor (VEGF) production and enhancing glycolytic activity. Furthermore, it significantly downregulated FABP4 expression, mitigating age-related metabolic dysfunctions, including hepatic steatosis, excessive fat accumulation, and senescence-associated secretory phenotype release. Notably, this design provides an alternative medication strategy for the elderly and addresses the shortcomings of the current market drugs, such as the frequency of administration, blood circulation time, and high toxicity and side effects. Specifically, the upgraded Pd/hCeO_2_ heterostructure exhibits rapid electron transfer at heterojunction interfaces, demonstrating superior multi-enzyme-like activity compared to CeO_2_, indicating enhanced ROS-scavenging capability and greater physiological regulatory potential [293]. Moreover, the PCBP system is administered via intravenous injection to improve drug bioavailability, enabling targeted drug delivery and the synergistic therapeutic effect of the Pd/hCeO_2_ heterostructure. Consequently, it effectively mitigates aging-related conditions at a substantially reduced dose.

Beyond the FABP family, CD36 represents another well-studied protein involved in lipid uptake and transport. As a scavenger receptor, CD36 is broadly expressed in multiple cell types, including macrophages, adipocytes, and muscle cells. This protein plays a crucial role in mediating the uptake of lipid species such as oxidized LDL and FAs via CD36-mediated endocytosis. In macrophages, CD36 is particularly necessary for regulating inflammatory responses and facilitating the clearance of apoptotic cells [294, 295]. Modulation of CD36 expression significantly influences both FA and cholesterol uptake [289]. In pathological macrophages, scavenger receptors like CD36 and lectin-like oxidized LDLR-1 play an important role in regulating oxidized LDL uptake, thereby attenuating macrophage foam cell formation. Conversely, inhibiting oxidized LDL internalization facilitates cholesterol balance and effectively suppresses plaque progression, making it a promising therapeutic strategy for AS [296]. Local lipid metabolic disorders, endothelial dysfunction, and progressive inflammation are key contributors to the pathogenesis of AS. Currently, AS is recognized as one of the most prevalent CVDs, posing a serious threat to human health, thereby underscoring the necessity for early diagnosis and intervention. Xu et al. [297] developed a pioneering theranostic (therapeutic + diagnostic) cascade-targeting nanoplatform, PA/ASePSD, which integrates astaxanthin and the mitochondria-targeted antioxidant SS31 peptide while incorporating the π-conjugated polymer PMeTPP-MBT as a photoacoustic contrast agent. The dextran shell exhibits a high affinity for Vascular Cell Adhesion Molecule-1 and CD44 on the ruptured endothelial surface, enabling PA/ASePSD to actively target AS lesions. Within the acidic plaque microenvironment, elevated ROS act as an intelligent cascade switch for the controlled release of astaxanthin, SS31 peptide, and PMeTPP-MBT. In vitro cellular uptake assays demonstrated that the SS31 peptide selectively targets macrophage mitochondria, downregulating CD36 and lectin-like oxidized LDLR-1 expression, thereby reducing ROS production, restoring mitochondrial function, and limiting cholesterol influx. Simultaneously, astaxanthin enhances ABCA1/G1 expression in foam cells, working synergistically with SS31 peptide to exert a potent anti-inflammatory effect. Furthermore, in vivo photoacoustic imaging revealed that the photoacoustic contrast agent PMeTPP-MBT enabled noninvasive real-time detection of early AS. In an ApoE^−/−^ mouse model of AS, PA/ASePSD generated strong photoacoustic signals at plaque sites, whereas signals were negligible in normal mice. Taken together, this synergistic strategy, integrating anti-inflammatory effects with the regulation of lipid metabolic homeostasis, exhibited robust anti-atherosclerotic efficacy and precise photoacoustic diagnostic capacity in in vitro and in vivo settings. This “three-in-one” therapeutic approach mediates lipid management through multiple pathways by reducing lipid uptake, promoting lipid efflux, and exerting anti-inflammatory effects [261]. In addition to using drugs to downregulate the expression of CD36, inducing its ubiquitination has also been illustrated as a feasible strategy. Liang et al. [262] discovered that intestinal-specific ACAT2 knockout mice could resist diet-induced obesity due to reduced intestinal lipid absorption. Simultaneously, combined with shACAT2 CaCO_2_ cells as an intestinal epithelial model, they revealed a novel mechanism by which intestinal ACAT2 regulates FA uptake-ACAT2 inhibition enhances the ubiquitination-mediated degradation of CD36 in intestinal cells, leading to a decrease in CD36 protein levels and a subsequent reduction in lipid absorption. Insufficient esterification of free cholesterol promotes ER stress and the recruitment of the E3 ligase RNF5, thereby activating CD36 ubiquitination in ACAT2-deficient intestinal cells. Therefore, they designed an ACAT2 siRNA/chitosan (CS)-poly(lactic-co-glycolic acid) (PLGA) NPs system, composed of PLGA-block-PEG to achieve intestinal delivery and ACAT2 inhibition. This system efficiently delivered ACAT2 siRNA specifically to the mouse small intestine and effectively suppressed intestinal lipid absorption, addressing obesity. NPs have previously been validated as suitable carriers for siRNA delivery in CRC therapy, wherein cells can internalize PLGA NPs, facilitating the release of functional siRNA into the cytoplasm [298]. The ACAT2 siRNA/CS-PLGA NP formulation utilizes the positive charge of CS to interact with the negatively charged intestinal mucosa, allowing its retention in the intestine and promoting siRNA delivery to intestinal epithelial cells. Thus, the therapeutic effect of ACAT2 siRNA inhibition in restricting intestinal FA absorption is attributed to the enhanced ubiquitination-mediated degradation of CD36 in intestinal cells. Notably, ACAT2 siRNA NPs do not enter the circulatory system, preventing unintended inhibition of hepatic ACAT2 activity, which could otherwise lead to TG accumulation [299].

SR-B1 is a scavenger receptor critically involved in cholesterol uptake, primarily facilitating cholesterol transport via interactions with HDL. This receptor plays an important role in reverse cholesterol transport, particularly in mediating cholesterol transfer from HDL in circulation to the liver [290]. Wang et al. [263] demonstrated that enhanced cholesterol uptake enables platinum-resistant (Pt-R) ovarian cancer cells to adapt to oxidative stress through lipid metabolic reprogramming, thereby fostering chemoresistance. This adaptive process is accompanied by the upregulation of glutathione peroxidase 4 (GPX4), an antioxidant enzyme crucial for mitigating oxidative damage. Given that Pt-R represents a major challenge in ovarian cancer treatment, leading to poor prognosis, developing effective therapeutic strategies is imperative. Subsequently, the researchers explored the interplay between cholesterol metabolism and redox equilibrium, proposing a dual-axis therapeutic strategy based on HDL NPs targeting the SR-B1 receptor. This approach impedes cholesteryl ester influx, enhances free cholesterol efflux, and triggers ferroptotic cell death, offering an innovative solution to overcome chemotherapy resistance. HDL NPs utilize 5 nm gold NPs as an inert core, providing structural stability and a platform for surface modification. The surface is functionalized with apolipoprotein A-I and a phospholipid bilayer, with an outer layer devoid of cholesterol, thereby maximizing the efflux of free cholesterol from the cell membrane. In vitro studies demonstrated that HDL NPs markedly suppressed cholesterol uptake, while simultaneously elevating LPO levels in Pt-R ovarian cancer cells. In vivo validation using a Pt-R ovarian cancer mouse model further confirmed that monotherapy with HDL NPs significantly reduced tumor burden and metastatic lesions. Moreover, the treatment exhibited excellent tolerability, as evidenced by the absence of weight loss. The specific synergistic therapeutic mechanism is divided into 2 parts: the bifunctional effect and epigenetic regulation. The bifunctional effect is exerted via 2 axes: Axis 1 (cholesterol deprivation) involves the inhibition of SR-B1-mediated cholesterol uptake, reduction of LDs formation, and disruption of membrane stability and energy metabolism; and Axis 2 (redox imbalance) involves the induction of LPO and ferroptosis by down-regulating the expression of GPX4, which shows a synergistic killing effect in combination with Pt-based drug. Meanwhile, epigenetic regulation involves indirect repression of SREBF2 by HDL NPs, leading to additional suppression of SR-B1 expression, thereby reinforcing a positive feedback loop. Cancer cells may evade treatment by upregulating other cholesterol uptake pathways or antioxidant pathways. Future approaches may require triple or even multi-target inhibition.

### Targeted intervention in lipid synthesis

Similar to lipid uptake, lipid synthesis is tightly orchestrated by the coordinated activity of multiple enzymes and proteins, including those involved in FA synthesis (FASN, ACC, SCD1, and ACLY) and cholesterol biosynthesis (HMGCR). Subsequently, a comparable carrier-based delivery strategy has been explored for targeted intervention in lipid synthesis. These approaches employ materials and therapeutic agents tailored to the disease microenvironment and can either trigger the downregulation of lipid synthesis-related genes or deliver specific inhibitors directly to target sites for precise gene suppression.

The accumulation of visceral fat is a primary driver of obesity, with both conditions mutually reinforcing each other in a self-perpetuating vicious cycle. However, effective targeted therapies for obesity remain elusive. In response, Wan et al. [264] pioneered the use of cationic polyamidoamine (PAMAM) dendrimers as a strategy to counteract obesity by modulating lipid synthesis and metabolism, thereby preventing adipocyte hypertrophy. Among the PAMAM dendrimers, polycation-based nanomedicine polyamidoamine generation 3 (P-G3) was selected due to its selective affinity for the highly anionic extracellular matrix (ECM), which is enriched with glycosaminoglycans in visceral fat. Following intraperitoneal injection, P-G3 preferentially accumulated in visceral fat rather than subcutaneous fat. Subsequent experiments in obese mice revealed that adipogenic differentiation genes (*Pparg*, *Cebpb*) exhibited normal expression during early differentiation, whereas key lipid synthesis genes (*FASN*, *SCD1*, *Srebf1*, and *DGAT2*) showed significant downregulation, leading to suppressed FA and TG synthesis. Consequently, adipocyte numbers were not affected; however, due to restricted lipid synthesis, they were unable to undergo hypertrophic expansion, ultimately forming metabolically healthier “dwarf adipocytes” [300, 301]. Additionally, in vitro adipogenesis models and single-cell RNA sequencing revealed that P-G3 mediates its effects through a coordinated mechanism, likely via the suppression of NAD and mTOR signaling pathways. This regulation uncouples lipid synthesis and storage from adipocyte development, resulting in functional adipocytes that do not undergo hypertrophic expansion. Researchers introduced 5 cholesterol-conjugated lipophilic chains to P-G3 via covalent linkage to further enhance targeting selectivity and minimize off-target effects. The modified P-G3-Chol (5) spontaneously formed cationic spherical NPs in water, providing a strong foundation for clinical translation. The cholesterol-modified P-G3 maintained a regulatory profile comparable to unmodified P-G3, but exhibited markedly reduced accumulation in the liver, kidneys, and lungs. This innovative paradigm introduces a new strategy for nanotherapeutics in metabolic diseases. Additionally, it holds potential for synergistic therapy through the co-delivery of lipid metabolism-modulating agents, such as FASN inhibitors.

Fucoxanthin (FX), a naturally occurring carotenoid, is a hydrophobic pigment known for its ability to regulate lipid metabolism, prevent obesity, and hold promise for the treatment of NAFLD. However, its intrinsic physicochemical drawbacks, including poor aqueous solubility and high susceptibility to oxidative degradation, pose major challenges for its biomedical application [302]. Wu et al. [265] designed a convenient orally administrable hepatic-targeted vesicle system encapsulating FX for targeted NAFLD therapy. The inner core of this carrier contains FX, the active ingredient for NAFLD treatment, protecting it from the harsh gastrointestinal environment while enabling precise in vivo delivery and enhancing its therapeutic efficacy. The outer structural component consists of biomimetic extracellular vesicles derived from Lactobacillus paracasei (LpEVs), with their surface functionalized with a targeting ligand, glycyrrhetinic acid (GA). This design not only protects the encapsulated FX but also utilizes the innate properties of LpEVs to evade immune clearance and ensure efficient delivery to target cells. Mechanistically, this FX-loaded nanocarrier system downregulated key lipid synthesis enzymes, including FASN, ACC1, and SREBP-1, in NAFLD model mice, thereby inhibiting lipogenesis, reducing fat accumulation, and slowing NAFLD progression. Subsequent study revealed that this system also improves hepatic levels of TG, FFAs, phosphatidylcholine, and SM in mice, thereby restoring lipid metabolism equilibrium.

HMGCR is a key enzyme in the cholesterol biosynthesis pathway, and its inhibition by statins, such as simvastatin (SIM), disrupts the mevalonate pathway, thereby reducing the synthesis of cholesterol and isopentenyl pyrophosphate (IPP). Given that IPP is essential for the post-translational modification of GPX4, its depletion directly impairs GPX4 function, leading to oxidative lipid damage and ferroptosis [303]. Building on this concept, Yao et al. [266] established a mechanistic link between cholesterol synthesis and ferroptosis by loading SIM onto Fe_3_O_4_ magnetic NPs functionalized with the zwitterionic polymer poly(carboxybetaine methacrylate) (Fe_3_O_4_@PCBMA) for the treatment of TNBC. The low pH and high glutathione (GSH) levels in the TME trigger the degradation of Fe_3_O_4_, leading to the release of Fe^2+^, which generates ROS via the Fenton reaction, along with SIM. The released SIM inhibits HMGCR, reducing downstream mevalonate pathway metabolites, such as IPP, resulting in GPX4 dysfunction, impaired lipid repair capacity, and LPO accumulation, ultimately inducing ferroptosis and establishing a comprehensive lipid metabolism regulatory axis. Notably, in vitro experiments indicated that HMGCR and GPX4 protein expression were significantly downregulated in the SIM-treated group, while LPO levels increased, confirming the inhibition of cholesterol biosynthesis, inactivation of GPX4, and accumulation of LPO. Subsequent in vivo studies demonstrated that Fe_3_O_4_@PCBMA-SIM markedly suppressed tumor growth while maintaining a favorable safety profile. Mechanistically, Fe_3_O_4_@PCBMA-SIM also targeted the HMGCR-MVA-GPX4 axis. Given metabolic adaptability, whether tumor cells can evade SIM inhibition by utilizing alternative lipid synthesis processes, such as DNL, remains unclear. Fe_3_O_4_ has been FDA-approved, and when combined with the repositioning of SIM, this strategy holds promising clinical translational potential.

Another intracellular enzyme, ACAT, has garnered interest in the regulation of cholesterol metabolism [45]. ACAT catalyzes the esterification of cholesterol by facilitating the conjugation of fatty acyl-coenzyme A with free cholesterol, thereby contributing to the regulation of intracellular cholesterol levels. ACAT activity increases with rising intracellular cholesterol levels, which promotes the conversion of free cholesterol into cholesterol esters for storage [304]. Conversely, ACAT activity is downregulated with a reduction of intracellular cholesterol levels, leading to a reduction in cholesterol ester formation, thereby maintaining cholesterol equilibrium within the cell [305]. Liu et al. [267] targeted ACAT-1 and incorporated photodynamic therapy (PDT) to engineer a tumor-penetrating nanovesicle system (namely EALP) for the co-delivery of avasimibe (AVA) and the photosensitizer phosphorbide A (PPa). These nanovesicles were constructed from lipid-based liposomes and functionalized with a matrix metalloproteinase-2 (MMP-2)-sensitive peptide, internalizing RGD (iRGD), and PPa, facilitating deep tumor penetration and precise drug release. Within the TME, MMP-2 enzymatically cleaves the peptide linker, releasing iRGD, which promotes tumor infiltration, while simultaneously triggering the co-release of AVA and PPa. As an ACAT-1 inhibitor, AVA increases membrane cholesterol levels, thereby enhancing T cell receptor signaling and cytokine secretion to boost T-cell function, and downregulates the expression of the cholesterol biosynthesis transcription factor SREBP2 and the migration-associated protein integrin αV, thereby suppressing tumor invasion. This provides an innovative metabolic intervention strategy for photodynamic immunotherapy.

ACC1 is the rate-limiting enzyme in FA synthesis, and its overexpression is critically associated with tumor progression. The transcriptional regulation of *ACC1* mRNA is driven by PPARγ activation, a process mediated by the deubiquitinating enzyme ubiquitin-specific protease 22 (USP22) [306]. Building on this mechanism, Zhao et al. [20] designed a dual-layered “gene + metabolic” inhibition strategy by integrating Clustered Regularly Interspaced Short Palindromic Repeats (CRISPR)-CRISPR-associated protein 9 (Cas9)-mediated *USP22* knockdown with ND630, a small-molecule ACC1 inhibitor, thereby simultaneously suppressing lipid synthesis at the transcriptional and enzymatic levels. This approach was implemented using a novel nanocarrier system, where red blood cell membrane (RBCM)-coated NPs were further functionalized with the tumor-targeting peptide glypican-3 (GPC3) to achieve precise tumor-targeted delivery. The inner core consists of ND630 encapsulated within a hydrophobic NTA-SS-PEG-SS-PCL core, forming micelles (NTA630). The intermediate layer comprises Cas9 complexed with small guide RNA, which is encapsulated within cationic acid-responsive nano-capsules (NCs), which bind to NTAG30 via electrostatic adsorption. Meanwhile, the outermost layer is an RBCM coating, which provides immune camouflage and extended circulation properties. The RBCM surface is further functionalized with the GPC3 targeting peptide, enabling specific binding to HCC cell surface markers, ultimately forming the novel nanocarrier system (NTA630-NCs-RBCM-T). Upon entering the TME, lysosomal acidification and intracellular high GSH levels trigger drug release. This personalized therapeutic strategy, combining gene editing and metabolic inhibition, significantly suppresses intracellular FA synthesis in cancer cells. Subsequent bioinformatics analysis revealed that, in addition to ACC1 and USP22, other classic lipid synthesis genes, including ACLY, FASN, and SCD, exhibited a PPARγ-dependent downregulation trend. Furthermore, the team discovered that this strategy disrupts lipid synthesis, leading to a reduction in SFAs, which in turn significantly enhances tumor cell uptake of PUFAs. This alteration triggers LPO, ultimately inducing tumor cell death via ferroptosis and apoptosis, demonstrating a potent antitumor effect and achieving a multi-modal cytotoxic mechanism. Accordingly, future studies hold promise for advancing tumor-specific peptide modifications, enabling broader applicability across various cancer types.

Similarly, Luo et al. [268] targeted lipid synthesis processes, selecting SCD1 as a therapeutic target. They are delivering an SCD1 inhibitor to cancer cells to modulate lipid metabolism, particularly to enhance ferroptosis, further highlighting the versatility and synergy of metabolic interventions in cancer therapy. However, ACC1 is located upstream of FA synthesis and regulates the conversion of acetyl-CoA to malonyl-CoA, whereas SCD1 is located in the FA desaturation link and directly affects membrane lipid composition and oxidative sensitivity. Luo et al. [268] developed a hypoxia-activated micelle (Aur/Plu@HM) containing nitroimidazole (NI), co-loaded with the SCD1 inhibitor PluriSin1 (Plu) and the ferroptosis inducer auranofin (Aur). In hypoxic TME, NI is converted into aminoimidazole (AI), facilitating drug release while simultaneously depleting antioxidant molecules. Additionally, AI exhibits stronger coordination with Zn^2+^ than histidine, enabling it to selectively strip SCD1 of its metal cofactor, thereby enhancing tumor cell sensitivity to Plu and Aur. Meanwhile, Plu directly inhibits SCD1 activity, whereas AI indirectly suppresses SCD1 via Zn^2+^ deprivation, thereby creating a dual blockade of lipid metabolic pathways. SCD1 inhibition reduces MUFA synthesis, increasing PUFA levels and promoting LPO accumulation. In contrast, Aur disrupts redox homeostasis by inhibiting thioredoxin reductase 1, leading to GSH and NADPH depletion, thereby amplifying ferroptotic signaling. Although this carrier design utilizes simple materials, its hypoxia responsiveness may be constrained by tumor heterogeneity.

Unlike the aforementioned strategies, Wu et al. [269] initially performed a targeted metabolomics analysis of plasma samples from 400 healthy individuals and patients with CRC, leading to the identification of matairesinol as a key differential lipid metabolite. Metabolic rescue experiments further confirmed that matairesinol levels were significantly depleted in patients with CRC compared to healthy individuals. Subsequent transcriptomic analysis revealed that matairesinol treatment resulted in pronounced suppression of TG synthesis-related genes, including PNLIP and DGAT2. Further mechanistic studies demonstrated that matairesinol supplementation disrupts lipid metabolism and attenuates ATP production, thereby inducing mitochondrial damage and oxidative stress in tumor cells. Subsequently, the researchers developed matairesinol-loaded liposomes (Liposome-Ma) and combined them with 5-Fu/leucovorin plus oxaliplatin (FOLFOX) to translate these findings into clinical applications. This combination restored chemotherapy sensitivity and significantly enhanced the antitumor efficacy of FOLFOX in CRC treatment, providing a novel therapeutic avenue for clinical CRC management [269].

### Targeted intervention in lipid storage and lipolysis

Under normal physiological conditions, LDs function as the principal lipid reservoirs and are increasingly recognized as central organelles in lipid metabolism. In recent years, LDs have emerged as a promising avenue in lipid metabolism research, with their potential applications in drug delivery garnering increasing interest [307]. Accumulating evidence suggests that tumor cells exhibit heightened metabolic activity, with markedly elevated LDs biogenesis relative to normal cells, highlighting LDs as potential targets for LDs-based therapeutic delivery [308]. Additionally, LDs inherently possess unique physicochemical properties, offering intrinsic potential as endogenous drug carriers [272].

Xu et al. [270] developed a biomimetic aggregation-induced emission (AIE) photosensitizer, DC@AIE dots, for LDs-targeted PDT. This approach integrates lipid metabolism regulation and immune activation, achieving a synergistic antitumor effect. Initially, the small-molecule AIE photosensitizer MeTIND-4 was identified for its exceptional NIR absorption and emission characteristics, as well as its optimal ROS generation efficiency. Characterized by a strong donor–acceptor structure, MeTIND-4 exhibits selective LDs affinity through a ‘like-dissolves-like’ interaction, driven by its intrinsic lipophilicity and the hydrophobic environment of the LDs core. Subsequently, given the critical role of DCs in immune responses, a cell membrane coating strategy was employed to encapsulate MeTIND-4 nanoaggregates with DC membranes, resulting in the formation of DC@AIEdots. Upon light irradiation, MeTIND-4 induces ROS production in LDs, triggering immunogenic cell death (ICD) and thereby activating antigen-presenting cells (APCs), while facilitating T-cell priming. DC@AIEdots retain DC membrane proteins and emulate the immunostimulatory role of APCs, potentiating CD4^+^/CD8^+^ T-cell activation and amplifying antitumor immunity. Colocalization experiments in 3T3-L1 cells revealed that the Pearson coefficient between the carrier and the LDs marker BODIPY was as high as 0.925, significantly exceeding those observed with other organelle markers. These findings demonstrate that MeTIND-4 is capable of penetrating cancer cells and specifically accumulating within LDs. In vivo, MeTTMN selectively accumulated in adipose LDs and induced Type I photodynamic lipid peroxidation upon light irradiation, ablating white adipocytes and promoting browning. This intervention markedly reduced body weight and fat mass in obese mice and improved glucose metabolism. This biomimetic delivery strategy leverages the external DC membrane to enable DC@AIEdots to engage in a ‘hitchhiking’ mechanism by attaching onto endogenous T cells, thereby boosting delivery efficiency by approximately 1.6-fold. In addition to its oncological applications, the team also investigated its expanded therapeutic potential and has reported two larger LDs-targeting aggregation-induced emissive luminogens (AIEgens) that utilize PDT-driven LPO for anti-obesity treatment [271]. A distinguishing feature of this approach is substituting AIE photosensitizers with hydrophobic TTMN and MeTTMN for targeting adipocytes. Notably, AIEgens selectively accumulate within the larger LDs of mature adipocytes, compared to smaller LDs in undifferentiated preadipocytes. Subsequently, MeTTMN generates ROS, exhibiting sustained ROS generation in hypoxic environments, rendering it well-suited for the hypoxic niche of adipose tissue. The primary advantage of the photosensitizer specifically targeting adipocytes in this study lies in its spatial controllability. It holds significant market potential not only for pathological obesity but also in medical aesthetics and body contouring. Treatment involves three variables: photosensitizer dosage, light exposure dosage, and individual fat thickness. Complex models must be developed to create personalized treatment plans for different patients and body regions, thereby preventing under-treatment or excessive damage.

As intracellular lipid reservoirs, LDs act as essential lipid storage units and double as drug carriers, facilitating the targeted transport of lipophilic drugs to cancer cells or adipocytes, thereby modulating lipid storage and lipolysis. The lipid matrix of LDs is intrinsically adapted for encapsulating lipophilic drugs and actively contributes to metabolic balance, while also demonstrating exceptional biocompatibility [309, 310]. Additionally, LDs can be readily isolated from other cellular components and are abundantly present in adipocytes, making them a promising candidate for scalable biomanufacturing. Furthermore, with advancing LDs research, the artificial synthesis of LDs is emerging as a viable and transformative strategy [307]. Consequently, Liang et al. [272] pioneered the engineering of modified LDs into multifunctional delivery systems, synergistically enhancing the efficacy of cancer PDT and propelling subcellular-targeted therapeutic strategies. In this study, 3T3-L1 cells, a widely recognized adipocyte differentiation model, were utilized to induce LDs differentiation and maturation. In vitro, 3T3-L1 preadipocytes underwent differentiation into mature adipocytes, and following LDs maturation, fully developed spherical LDs were isolated. The size and monodispersity of the formed LDs can be fine-tuned by modulating insulin concentration, where a shorter insulin exposure time improves LDs homogeneity. The photosensitizer pyropheophorbide-a was chemically conjugated to lipid molecules via covalent bonding, forming Pyrolipid. Following 24 h co-incubation with differentiated adipocytes, Pyrolipid was selectively enriched within LDs through lipid affinity-driven self-loading, leading to the generation of Pyrolipid@LDs. After freeze-drying, these LDs exhibited long-term stability under storage and, upon reconstitution, maintained their intact structure and sustained drug-loading capacity. Subsequent biological assays confirmed that laser activation of Pyrolipid@LDs led to ROS generation, initiating LPO. Additionally, the lipid matrix of LDs served as an oxidation substrate for ROS, exacerbating lipotoxicity, perturbing metabolic balance, and triggering apoptosis in cancer cells. Thus, in this study, LDs served as drug reservoirs and metabolic modulators, effectively integrating PDT with metabolic regulation, thereby circumventing the hypoxia-associated constraints of conventional treatments.

Currently, adipocyte lipolysis has been extensively studied in the context of lipid metabolism, especially since the onset of metabolic disorders like obesity is often linked to a decline in fat breakdown rates, contributing to excessive lipid accumulation and obesity progression. Particularly, ATGL, HSL, and MGLL are recognized as important regulators of lipolysis [102]. Therefore, lipolysis-activating pharmacological agents or interventions could facilitate fat mobilization and reduce lipid accumulation in obesity management. For instance, specific bioactive molecules can enhance the enzymatic activity of ATGL and HSL, thereby accelerating lipid hydrolysis and subsequent energy utilization [311]. Nevertheless, existing pharmacological interventions for obesity result in significant side effects, thereby restricting their clinical applicability and increasing the risk of comorbidities [312]. This highlights the urgent need to explore innovative therapeutic alternatives beyond conventional treatments. Subsequently, Wang et al. [273] explored and harnessed the potential of plant-derived nanovesicles, which offer natural origin, superior biocompatibility, efficient lipophilic bioactive encapsulation, and precise targeting capabilities. Building upon these advantages and capitalizing on the multifunctional metabolic modulation potential of turmeric, a bioinspired nano-formulation was developed: Reconstructed Turmeric-derived Nanovesicles (Rec-tNVs). PEG precipitation was utilized for the initial enrichment of curcumin nanovesicles, yielding ultracentrifugation-isolated tNVs (UC-tNVs). These nanovesicles were reassembled into Rec-tNVs using solvent extraction and thin-film hydration techniques. Quantitative nano-flow cytometry was employed to quantify the curcumin content per Rec-tNV, revealing that Rec-tNVs exhibited a curcumin concentration four orders of magnitude greater than traditional UC-tNVs. In vitro studies using 3T3-L1 adipocytes revealed that Rec-tNVs exert a dose-dependent lipid-lowering effect. Mechanistically, this effect is attributed to the stimulation of lipolysis, supported by the upregulation of lipolytic enzyme genes Atgl and Hsl, alongside the downregulation of adipogenesis-related genes Fabp4 and Dgat. These findings indicate that Rec-tNVs promote lipolysis in adipocytes and suppress adipogenesis. In addition, Rec-tNVs enhance lipase activity, upregulate UCP1 expression, and facilitate adipocyte browning. At high doses, Rec-tNVs further induce apoptosis in adipocytes through mitochondrial depolarization and caspase-3 activation. In vivo studies provided further evidence supporting the efficacy of Rec-tNVs, highlighting their potential roles in lipid balance, hepatoprotection, adipose remodeling, and gut microbiome modulation. Thus, Rec-tNVs address the intrinsic limitations of curcumin and offer a multi-target intervention strategy for obesity treatment by applying their natural origin and superior drug-loading capacity. In the future, this reconstructed nanovesicle could serve as a universal delivery platform capable of not only carrying curcumin's active components but also co-loading other anti-obesity drugs. This approach achieves synergistic effects between “natural multi-effect therapy” and “targeted potent therapy”, potentially reducing required drug doses and side effects.

Advances in genetic engineering have led to rapid advancements in gene editing and vector-based lipid metabolism modulation. Previous study demonstrated that miR-130b mitigates lipid accumulation via PPAR-γ targeting; however, unprotected miRNA is highly susceptible to degradation by circulating RNases, highlighting the necessity of protective delivery vectors [313]. Pan et al. [274] introduced microvesicles (MVs) as natural carriers, which can bypass immune surveillance, shield miRNA against enzymatic degradation, and efficiently deliver it to target tissues. A miR-130b overexpression plasmid was introduced into HeLa-229 cells via transfection to generate miR-130b-enriched microvesicles (miR-130b-MV), and the secreted MVs, highly enriched in miR-130b, were isolated. Mechanistic studies further revealed that miR-130b-MV upregulated the mRNA expression of key lipolytic enzymes, including HSL and MGLL, while exerting no significant impact on the mRNA levels of FASN and ACC. These findings suggest that miR-130b-MV primarily facilitates fat reduction by enhancing lipolysis through translational repression of PPAR-γ rather than inhibiting lipid biosynthesis. While this therapeutic approach demonstrates short-term safety and efficacy, its long-term outcomes, miRNA half-life, and potential off-target effects on other tissues necessitate further exploration.

### Targeted intervention in lipid oxidation

Under elevated energy requirements, such as during prolonged exercise or fasting, adipose TGs are hydrolyzed into FAs and glycerol. The released FAs are transported to the liver and other tissues, where they undergo oxidation to fuel essential physiological processes, ensuring cellular homeostasis [102]. In a healthy liver, hepatic lipid metabolism maintains intracellular lipid equilibrium, while controlled lipid oxidation supports hepatic energy metabolism, sustaining normal liver function [314]. However, under pathological conditions, this delicate balance is disrupted in hepatocytes, predisposing the liver to metabolic disorders and associated diseases. A previous study indicates that dysfunctional hepatocytes generate excessive ROS, surpassing the capacity of the endogenous antioxidant system. This imbalance induces oxidative stress, which in turn accelerates inflammation and fibrosis progression [315]. At this stage, rapid ROS elimination can interrupt the pathological feedback loop, preventing hepatic fibrosis and necrosis. While overactive antioxidant therapy poses potential risks, prolonged ROS suppression may impair abnormal cell clearance, potentially facilitating tumorigenesis [316]. Additionally, complete ROS elimination could hinder liver regeneration and antiviral immune responses. In contrast, retaining physiological ROS at low levels is considered beneficial for cellular adaptation, as moderate ROS induces nuclear factor erythroid 2-related factor 2 (Nrf2)-mediated antioxidant enzyme expression, enhancing cellular resilience against oxidative stress [135]. The former approach is suitable for acute oxidative stress-induced injury, where rapid ROS elimination blocks pathological progression, whereas the latter applies to chronic or recovery phases, where ROS homeostasis supports physiological function and promotes tissue regeneration. Hence, a precision ROS modulation strategy, tailored to etiology, disease stage, and individual antioxidant capacity, may achieve damage mitigation and regenerative support. Accordingly, recent advances in nanocarrier design have focused on two distinct intervention strategies for lipid oxidation: 1) scavenging pathological ROS and 2) preserving physiological ROS, both of which have yielded encouraging results in lipid oxidation regulation.

The selective ROS-elimination approach has therapeutic potential for a wide range of diseases, including liver disorders, OA, and autoimmune or inflammatory conditions [317]. In the pathological context of liver fibrosis mentioned above, profibrotic factors released by activated hepatic stellate cells (HSCs) can induce capillarization of liver sinusoidal endothelial cells (LSECs), in addition to excessive ROS production. This impedes gas exchange in the liver microenvironment, inhibits NO production, causes hypoxia, and disrupts lipid metabolism, particularly the FAO, thus aggravating hepatocyte dysfunction [318]. Based on this, Zhang et al. [275] designed 2 lipid NPs, namely chondroitin sulfate-modified vismodegib-loaded NPs (CS-NPs/VDG) and glycyrrhetinic acid-modified silybin-loaded NPs (GA-NPs/SIB), which can target the repair of LSECs, inhibition of HSCs, and restoration of hepatocyte function, ultimately reversing liver fibrosis by regulating lipid oxidation and metabolic pathways. CS-NPs/VDG is a vismodegib (VDG)-loaded NP modified by CS. CS enables dual-targeting of these NPs by binding to the Stabilin 2 receptor on LSECs and the CD44 receptor on HSCs. Meanwhile, drug-loaded VDG, a Hedgehog signaling pathway inhibitor, can repair the fenestrae phenotype of LSECs, restore liver sinusoid permeability, enhance NO production, and improve the β-oxidation capacity of hepatocyte FAs. In addition, drug-loaded VDG reduces HSC activation and the release of profibrotic factors, indirectly improving the lipid metabolic microenvironment. GA-NPs/SIB is a GA-modified SIB NP, wherein GA enhances targeting efficiency by binding to the high-expression GA receptors on hepatocytes. Additionally, the drug-loaded SIB alleviates oxidative stress by scavenging ROS and can repair hepatocyte function and promote regeneration. Following combined treatment, LSECs restored normal NO secretion and promoted the quiescence of HSCs, which in turn degraded the ECM, alleviating hepatocyte injury. Meanwhile, restored hepatocytes secreted VEGF to sustain the LSEC phenotype, establishing a virtuous circle. This design innovatively proposed the approach of treating liver fibrosis by regulating multicellular interaction networks, overcoming the limitations of traditional single-target strategies, and offering a novel therapeutic avenue for chronic liver diseases associated with metabolic disturbances.

Moreover, in the context of metabolic diseases such as OA, Wang et. al [276] collaborated to develop a multi-target NP (Ce@D&P NPs), with lipid oxidation and lipid metabolism regulation as the core therapeutic mechanism. Deferasirox (DEF), an iron chelator, binds to Fe^3+^ and inhibits iron-dependent LPO, while pterostilbene has anti-inflammatory and antioxidant effects and can repair the cartilage matrix. The 2 compounds are covalently linked via a thioether linker to form the bifunctional molecule D&P. The incorporation of cerium (Ce) NPs compensates for potential ROS elevation induced by DEF, enhancing the antioxidant capacity of the system. Transcriptomic and metabolomic analyses showed that Ce@D&P NPs significantly regulated the expression of genes related to oxidative stress and lipid metabolism in chondrocytes stimulated by IL-1β, exerting anti-ferroptotic and anti-inflammatory effects via pathways such as PI3K/Akt and nuclear factor kappa-B. Furthermore, metabolomic analysis revealed the regulatory effects of the NPs on chondrocyte metabolism, including the modulation of FAO, lipid metabolism, and antioxidant metabolic pathways. This multi-omics approach provides a comprehensive perspective on the molecular mechanism of nanomedicine. In vivo results from the DMM-induced OA mouse model further confirmed that Ce@D&P NPs significantly alleviated cartilage damage, reduced osteophyte formation, and regulated lipid metabolism-related markers through intra-articular injection. In this study, multiple metabolic pathways were employed to reverse the metabolite change caused by IL-1β stimulation, providing important directions for understanding the molecular biological mechanisms of nanomaterials in inhibiting chondrocyte ferroptosis under disease conditions [276]. Ferroptosis also plays a crucial role in other cartilage-related diseases, such as cartilage destruction in rheumatoid arthritis and intervertebral disc degeneration. Nanotechnology platforms that have been successfully applied to OA could, after appropriate modification, be repurposed for the treatment of these diseases, offering enormous market potential and broad application prospects.

Similarly, Cai et al. [277] explored crosstalk between hepatic lipid metabolism and oxidative processes, developing a low-dose NP system to induce a controlled ROS increase, an approach that contrasts with Zhang et al. [275], who focused on ROS elimination to mitigate hepatocyte injury. This approach activated antioxidant responses and lipolytic enzymes instead of inducing pathological oxidative stress, thereby enhancing beneficial lipid metabolism. Consequently, this study provides a new perspective on the dual role of lipid oxidation in metabolic regulation. Considering that nanocarrier materials must possess low cytotoxicity, low biodegradability, and high biocompatibility, TiO2, Au, and NaYF4 were selected as inorganic NP candidates. The NP size was meticulously regulated between 14 and 17 nm, as smaller dimensions enhance hepatocyte uptake efficiency. The results showed that low-dose [0.72 mg/(kg·d)] NPs induced a mild increase in ROS levels within hepatocytes, subsequently activating the antioxidant transcription factor Nrf2. Activated Nrf2 binds to the antioxidant response element in the promoter region of Ces2h, leading to a significant upregulation of Ces2h expression. Ces2h plays a dual role. As a carboxylesterase, Ces2h facilitates the hydrolysis of TG and CE, thereby liberating FFA. The released FFA activates PPARα, which triggers FAO and enhances the transcription of FAO-associated genes, including *Cpt1b* and *Acox1* [319]. Throughout this process, the NPs exert minimal influence on lipid synthesis or VLDL secretion. Instead, they exclusively modulate oxidative and lipolytic pathways. Conversely, Ces2h enzymatically neutralizes hydroxyl radicals, thereby preserving cellular redox homeostasis. Notably, during NP safety assessments, high doses [18 mg/(kg·d)] or Ces2h deficiency could induce oxidative stress and toxicity. Consequently, these results underscore the necessity of dose control and highlight the need to consider the impact of genetic variability. Overall, this study innovatively provides evidence that NPs regulate lipid oxidation via the Nrf2-Ces2h pathway, circumventing conventional antioxidant enzymatic pathways. This phenomenon stems from the dual function of Ces2h, which serves as a lipolytic enzyme and a ROS buffer, thereby orchestrating metabolic equilibrium and oxidative stress regulation. These findings emphasize the extensive metabolic regulatory potential of inorganic NPs, positioning them as promising candidates for future clinical applications in NAFLD and obesity treatment.

A similar therapeutic approach was independently investigated by Zhou et al. [278], who developed controlled-release chenodeoxycholic acid-loaded gelatin NPs (CDCA-GNPs), delivered via injection, as a potential therapy for obesity. Utilizing CDCA, an endogenous bile acid, this system activates the nuclear receptor farnesoid X receptor (FXR) and the membrane receptor Takeda G protein-coupled receptor 5 (TGR5), consequently facilitating FAO, lipolysis, and mitochondrial bioenergetics [320, 321]. Mechanistically, CDCA-GNPs significantly upregulated FAO-related genes (Ppara, Cpt1b), lipolysis-associated genes (Atgl, Hsl), and mitochondrial function regulators (Ucp1, Prdm16). Moreover, brown adipocytes released FFAs via lipolysis, further triggering UCP1 activation in a feedforward manner, thereby enhancing thermogenesis and energy expenditure. Distinct from the strategy employed by Cai et al. [277], CDCA-GNPs further induced a “futile cycle”, wherein simultaneous lipolysis and lipogenesis dissipate energy, amplifying fat oxidation in white adipocytes. Gelatin, a biodegradable and biocompatible polymer, was employed as the nanocarrier matrix, while β-cyclodextrin was used to improve hydrophobic drug loading and optimize delivery. The sustained local release of CDCA-GNPs minimized their systemic exposure, reducing adverse effects associated with FXR/TGR5 systemic activation. Accordingly, CDCA-GNPs are a promising alternative to synthetic sodium deoxycholic acid, which is associated with inflammation, bruising, and necrosis. However, reduced patient adherence compared to oral administration of CDCA-GNPs may impact its clinical translation. If the challenges of systemic drug delivery can be overcome, it holds promise to become one of the cornerstone therapies for treating a range of metabolic disorders such as obesity, NAFLD, and diabetes. Particularly when combined with existing medications, it could provide patients with multi-pathway, precision metabolic health management solutions.

In cholesterol metabolism, COD serves as an important enzyme catalyzing cholesterol oxidation. Under aerobic conditions, COD facilitates the oxidation of the 3-hydroxyl group of cholesterol into a keto group, yielding cholest-4-en-3-one and concurrently generating H_2_O_2_ [123]. Du et al. [279] used H_2_O_2_ to construct a metal-organic framework (MOF)-based COD immobilization platform, encapsulated within a chondroitin sulfate gel shell (DOX@MOF-COD@CS). The MOF framework exhibits peroxidase-mimetic activity, catalyzing the conversion of H_2_O_2_ into hydroxyl radicals (·OH), thereby exerting direct cytotoxic effects on tumor cells and implementing a waste-to-resource therapeutic approach. Moreover, CS enhances tumor targeting via CD44 receptor interaction and enables GSH-responsive drug release, thereby enhancing multimodal therapeutic efficacy and reversing multidrug resistance. However, in cancer therapy, the hypoxic microenvironment often compromises COD functionality, while cholesterol depletion induces a pro-survival autophagic response, leading to treatment resistance. These challenges pose significant barriers to the clinical translation of COD-based cholesterol metabolism-targeted cancer therapies [322, 323]. Therefore, Zhang et al. [280] developed a COD-integrated MOND system to overcome these limitations. Molybdenum oxide (MoO3-x) nanodots were selected due to their Mo5^+^/Mo6^+^ valence states, which facilitate electron transfer to enhance H_2_O_2_ decomposition activity, thereby catalyzing O_2_ generation. By supplying O_2_ to COD, this system alleviates tumor hypoxia, enhances COD activity, and establishes a self-sustaining cholesterol oxidation cycle through a positive feedback loop between H_2_O_2_ and O_2_. Furthermore, MONDs activate Akt/mTOR phosphorylation, thereby suppressing autophagy and disrupting tumor protective mechanisms.

However, the therapeutic potential of COD extends beyond localized treatment. Shen et al. [281] further investigated its role in mitigating postoperative tumor recurrence and metastatic progression, aiming to improve poor prognostic outcomes. Following microwave ablation (MWA) of HCC, cholesterol-enriched tumor debris facilitates the establishment of an immunosuppressive residual tumor microenvironment (IRTM), driving elevated recurrence and metastatic progression. This study introduced a cholesterol-targeting catalytic hydrogel (DA-COD-OD-HCS) to address this challenge, which is capable of catalyzing cholesterol degradation and triggering ferroptosis, thereby modulating IRTM dynamics and potentiating the therapeutic synergy between MWA and immune checkpoint inhibitors. In this system, dimethylmaleic anhydride undergoes activation within the acidic TME, leading to pH-responsive COD release. Additionally, hemin-CS sulfate, functioning as a Fenton reaction catalyst, amplifies hydroxyl radical generation, enhancing oxidative damage. Meanwhile, oxidized dextran and HCS form a dual-network hydrogel, effectively extending the in-situ retention of COD and hemin for prolonged therapeutic action. Thus, upon intratumoral injection, DA-COD-OD-HCS applies tumor debris as a metabolic substrate, continuously catalyzing cholesterol degradation and lipid oxidation, ultimately remodeling the IRTM and eliciting systemic antitumor immunity. This study proposes a cholesterol-targeted catalytic hydrogel that ingeniously integrates localized physical therapy, chemokinesis therapy, metabolic therapy, and immunotherapy into a self-reinforcing antitumor “virtuous cycle”. Its therapeutic potential extends far beyond serving merely as an adjunct to microwave ablation; it holds promise to evolve into a platform-based core strategy for tumor immunotherapy.

### Targeted intervention in lipid metabolic reprogramming

Lipid metabolism reprogramming is intricately linked to tumor malignancy, governing key processes in tumor and immune cells, albeit with distinct mechanisms and implications in each cell type. In tumor cells, lipid metabolism reprogramming primarily fuels rapid proliferation and metastatic potential, confers apoptosis resistance, and influences the TME. In contrast, in immune cells, it orchestrates immune activation and functionality, regulates differentiation and persistence, and reshapes the TME landscape [100]. Thus, lipid metabolism reprogramming exemplifies the adaptability of lipid metabolic pathways, positioning it as a crucial target for tumor prevention, early diagnosis, and innovative therapeutic exploration. Consequently, considering the intricate interplay within metabolic networks, including the metabolic crosstalk among normal, tumor, and immune cells, the dynamic impact of the TME, and the challenges associated with targeted metabolic intervention, harnessing drug delivery technologies and advanced biomaterials to precisely reprogram lipid metabolism emerges as a promising strategy for combating cancer and metabolic diseases.

Accordingly, Xie et al. [282] developed a nanotherapeutic platform integrating Fe_3_O_4_ NPs and 1H-perfluoropentane (1H-PFP), coated with PLGA-PEG and functionalized with a prostate cancer-targeting heterodimeric polypeptide (GBP@Fe_3_O_4_) to modulate lipid metabolic reprogramming in tumor cells. Fe_3_O_4_ NPs possess superior biocompatibility and potent Fenton catalytic properties, promoting the robust production of ROS. Additionally, 1H-PFP experiences a thermally induced liquid-to-gas phase transition, enabling the swift dispersion of NPs. In this study, GBP@Fe_3_O_4_ triggered the prompt release of Fe_3_O_4_ NPs upon exposure to controlled thermal stimulation at 45 °C, facilitating spatiotemporal control over ROS production within the TME. Concurrently, it inhibited cellular antioxidant defense mechanisms, exacerbating oxidative stress and culminating in ferroptotic cell death. Driven by the synergistic effects of thermal stress and Fe_3_O_4_ NPs, lipid metabolic pathways in tumor cells underwent significant reprogramming, resulting in excessive LPO accumulation. Subsequent RNA sequencing of C4-2 prostate cancer cells treated in vitro revealed substantial gene expression shifts in lipid metabolism affecting the biosynthetic pathways of PUFAs, which are necessary for ferroptosis susceptibility. Furthermore, RNA sequencing and Western blotting analysis identified a significant upregulation of acyl-CoA synthetase bubblegum family member 1 during this process, implicating it as an important regulator of ferroptosis. This discovery was further validated in vivo, where upregulation of ACSBG1 expression was observed in C4-2 tumor-bearing mice following GBP@Fe_3_O_4_ heat treatment and opens new avenues for potential clinical translation. This strategy modulates tumor cell death pathways via externally applied thermal stress and, when integrated with tumor-specific metabolic reprogramming, facilitates a controlled shift from ferroptotic to non-ferroptotic cell death. This circumvents the systemic toxicity frequently observed in conventional therapies, providing a highly targeted and safer therapeutic alternative. A major challenge for clinical translation lies in how to confine ferroptosis strictly within the tumor site, thereby preventing damage to normal tissues, particularly organs rich in PUFAs and iron, such as the liver and kidneys.

Xu et al. [324] engineered an NP platform with selective affinity for tumor-associated dendritic cells (TADCs) to reprogram lipid metabolism in TADCs. This system modulates multiple lipid metabolic pathways simultaneously, effectively restoring TADC functionality. Lipid accumulation in TADCs plays a central role in their dysfunction, encompassing exogenous lipid uptake, DNL, and transcriptional activation of lipogenic genes [137]. Accordingly, engineered NPs were designed to selectively regulate multiple lipid metabolic pathways. These NPs are loaded with 5-(tetradecyloxy)-2-furoic acid (TOFA) and STF-083010 (STF). TOFA inhibits ACC, the rate-limiting enzyme in FA synthesis, thereby reducing intracellular triacylglycerol (TAG) accumulation, whereas STF, an XBP1 inhibitor, attenuates ER stress-induced upregulation of lipogenic genes. Moreover, the NP exterior is functionalized with the Msr1 antagonist fucoidan (FU), which acts as a competitive inhibitor of Msr1-mediated lipid uptake in TADCs. This multifunctional NP formulation is designated as TS-PP@FU. In vitro studies illustrated that PP@FU markedly attenuated lipid deposition in DCs treated with PA in a dose-dependent manner, leading to the effective blockade of lipid uptake. Additionally, T-PP NPs significantly reduced intracellular TAG accumulation without affecting cell viability at optimal doses, suggesting an inhibition of FA synthesis. Meanwhile, S-PP NPs downregulated ER stress-responsive and lipogenic gene expression in PA-stimulated DCs, confirming transcriptional repression of the lipid synthetic pathway. Furthermore, TS-PP@FU completely reversed PA-mediated suppression of lipopolysaccharide-induced DC maturation markers. Accordingly, this strategy effectively reinstated TADC antigen-presenting function through a multi-tiered intervention targeting lipid uptake, synthesis, and transcription across extracellular, cytoplasmic, and nuclear compartments, thereby promoting CD8^+^ T-cell recruitment and activation. Consistent with in vitro findings, in vivo studies revealed that TS-PP@FU, in combination with anti-programmed death-1, markedly inhibited tumor progression, reversed the immunosuppressive TME landscape, and facilitated robust CD8^+^ T-cell infiltration, successfully transforming “cold tumors” into anti-programmed death-1-responsive “hot tumors”. This study emphasizes the versatility of multi-target metabolic modulation, indicating its potential applicability across lipid-rich malignancies, including ovarian cancer and melanoma.

In contrast, Qin et al. [283] reprogrammed TADC lipid metabolism by applying NPs to revert their immunosuppressive phenotype, thereby potentiating anti-tumor immunity. Their approach adopted a single-target inhibition strategy, primarily targeting FA synthesis inhibition through TOFA, while their design focused on alleviating oxidative lipid accumulation, a key factor impeding antigen cross-presentation, and placed greater emphasis on the adaptability of in situ vaccination and durable immune memory formation. Tumor-infiltrating DCs undergo aberrant lipid accumulation, resulting from disrupted lipid balance in the TME, thereby compromising their antigen cross-presentation efficiency. Hence, NPs were designed to deliver the FA synthesis inhibitor TOFA, thereby inhibiting DNL and mitigating intracellular lipid overload. Particularly, the NP exterior was functionalized with bacterial outer membranes (OMs) and DSPE-PEG2000-MAL, enabling the selective capture of tumor-associated antigens through a Michael addition reaction. Furthermore, pathogen-associated molecular patterns facilitated DC-selective targeting, culminating in the development of a NP-based vaccine formulation, TOFA@PLGA@OMs-PLs. This strategy synthesizes bacterial OM-mediated targeting, antigen sequestration, and lipid metabolic intervention, introducing a pioneering “capture-delivery-metabolic reprogramming” paradigm. The 2 studies offer complementary approaches to reprogram DC metabolism in tumor immunotherapy, which are centered on holistic metabolic modulation and integrated antigen-metabolism synergy, respectively. Together, they can propel advancements in DC-targeted metabolic interventions, paving new avenues for tumor immunotherapy.

Additionally, strategies for T-cell lipid metabolism reprogramming aim to overcome the metabolic constraints imposed by glucose scarcity in the TME, which leads to T-cell dysfunction and reduced anti-tumor potency [325]. Metabolic plasticity can be reinstated by harnessing FAs as an alternative fuel, which can, in turn, augment effector responses. Accordingly, Yu et al. [284] developed a CD3/F/AN NPs for the targeted delivery of metabolic modulators, specifically adapted for low-glucose, high-lactate, metabolically restrictive TMEs. This system, built on amphiphilic poly(γ-glutamic acid) NPs, serves as a carrier for the PPARα agonist fenofibrate, thereby facilitating the upregulation of FAO-associated genes. The anti-CD3ef(ab’)2 antibody fragments were immobilized onto the NP surface, exploiting CD3 receptor-mediated uptake to optimize T-cell specificity, ultimately augmenting T-cell viability and tumoricidal function. Subsequent in vitro and in vivo studies confirmed that PPARα activation led to the upregulation of key FAO genes, including CPT1B, LCAD, and MCAD, thereby shifting metabolic dependency toward FAO. Additionally, the TME underwent significant remodeling, resulting in a 14.8-fold enhancement in T-cell survival and proliferation in vivo and a 5.4-fold increase in vitro. The expression of effector molecules granzyme B and IFN-γ exhibited substantial upregulation in tumor tissues and infiltrating T cells, suggesting potentiated anti-tumor cytotoxicity. Thus, the study integrates metabolic regulation with immune modulation, utilizing nanotechnology to rewire T-cell lipid metabolism, and highlights the promise of metabolic immunotherapy as an emerging therapeutic paradigm.

Lipid metabolism reprogramming in TAMs enables bidirectional modulation of their pro-inflammatory (M1) and anti-inflammatory (M2) phenotypes, thereby tailoring immune responses to distinct disease contexts and highlighting the adaptability of metabolic interventions [326]. In cancer, TAMs are primarily skewed toward the immunosuppressive M2 phenotype; therefore, shifting their polarization toward the pro-inflammatory M1 phenotype is a promising strategy to potentiate anti-tumor immunity. Accordingly, Cao et al. [222] engineered a redox-responsive RNAi-based nanoplatform, enabling the co-delivery of siMGLL (monoacylglycerol lipase siRNA) and siCB-2 (endocannabinoid receptor-2 siRNA). This dual-pronged approach concurrently disrupts lipid metabolism in tumor cells and drives TAM polarization toward an M1 phenotype, culminating in a synergistic anti-cancer therapeutic effect. CB-2 is overexpressed in TAMs, where its activation by the 2-arachidonoylglycerol (2-AG) axis reinforces the M2 phenotype. In this study, the central mechanism underlying TAM lipid metabolic reprogramming relies on NP-mediated targeted siCB-2 delivery to TAMs, which silences CB-2 expression, disrupts the 2-AG signaling cascade, and redirects TAM polarization toward the pro-inflammatory M1 phenotype, ultimately restoring anti-tumor immune responses. However, in tumor cells, MGLL suppression results in excessive 2-AG accumulation, which can exert paracrine effects on CB-2 receptors in TAMs, potentially offsetting the therapeutic advantages of single-target intervention. In this regard, the co-delivery of siMGLL and siCB-2 allows concurrent inhibition of tumor cell-derived FFA synthesis and suppression of M2-polarizing signaling in TAMs, thus preventing the immunosuppressive effects of metabolic byproducts within the TME. The core of the NP platform consists of reduction-responsive polydisulfide amide and cationic lipid, which efficiently encapsulates siRNA. The outer shell, which is composed of 1,2-Distearoyl-sn-glycero-3-phosphoethanolamine-N-[methoxy (polyethylene glycol)-3000], enhances prolonged blood circulation and tumor targeting. In the future, by assessing the proportion of M2-type macrophages and the specific lipidomic profile in a patient’s tumor tissue, it may be possible to identify those most likely to benefit from this therapy, thereby achieving truly personalized precision medicine.

In contrast, inflammation-associated pathologies, such as OA and NAFLD, feature excessive M1 macrophage infiltration and polarization, which drive the onset and chronicity of low-grade inflammation in joint degenerative disorders such as OA [327]. Similarly, NAFLD arises from a convergence of lipid metabolic dysregulation and chronic inflammation, wherein M1 macrophages intensify insulin resistance and lipid imbalance via pro-inflammatory cytokine secretion [328]. In both conditions, TAMs are largely skewed toward the inflammatory M1 phenotype, and lipid metabolic reprogramming fosters M1-to-M2 repolarization by modulating compensatory pathways governing inflammation and oxidative stress, ultimately mitigating inflammatory burden [329]. Accordingly, Deng et al. [285] engineered a Golgi-targeted nanodrug, a self-assembled nanocarrier (designated as CSBN), based on a chondroitin sulfate-bilirubin (CS-BR) conjugate for the targeting delivery of licofelone (LCF), LCF-CSBN. CSBN is capable of dual regulation of sphingolipid and arachidonic acid metabolism, thereby promoting M1-to-M2 macrophage repolarization and mitigating OA-induced inflammation and cartilage degeneration. In M1 macrophages, the Golgi apparatus undergoes pathological swelling, characterized by Golgi stress, further perturbing lipid balance and triggering excessive ROS buildup. LCF-CSBN consists of CS and BR conjugated via an ethylenediamine linker, yielding the CS-BR macromolecular complex. CS selectively binds to CD44 receptors, which are overexpressed in M1 macrophages, whereas the hydrophobic BR core ensures ROS sensitivity, facilitating intracellular ROS neutralization and oxidative stress attenuation. This amphiphilic polymer spontaneously forms CS-BR NPs, prolonging intra-articular retention for up to 28 d, thereby minimizing administration frequency. Upon intra-articular injection, LCF-CSBN precisely accumulates within the Golgi apparatus, restoring Golgi integrity by reverting its swollen morphology to a flattened vesicular state. The encapsulated COX-2/5-LOX dual inhibitor, LCF, inhibits the arachidonic acid metabolic pathway, acting in synergy with BR to regulate lipid metabolism and rewire macrophage lipid balance.

Optimizing linker arm flexibility and length to refine targeting precision or incorporating anti-inflammatory and pro-repair agents to strengthen M2 polarization represents another promising avenue for further refinement. Wang et al. [286] refined this approach for NAFLD therapy by incorporating charge-reversal mechanisms to concurrently modulate macrophage-mediated inflammation and hepatocyte lipid balance, thus addressing the constraints of single-target interventions. Dimethylmaleic anhydride (DMA) was incorporated as an acid-sensitive functional group, which was covalently linked to polyetherimide (PEI) through amide bonding, effectively masking the inherent positive charge of PEI and extending its systemic circulation time. In acidic, inflammatory, and lysosomal environments, the amide bond of DMA hydrolyzes and detaches, leading to charge reversal, which facilitates preferential macrophage uptake. Upon crossing the cell membrane, the NPs accumulate in mitochondria, enhancing local drug concentration. Additionally, fullerene poly (ethylene glycol) molecules, acting as highly efficient ROS scavengers, self-assemble with PLGA through hydrophobic interactions, forming FPPD NPs. Thus, FPPD exerts dual effects of macrophage reprogramming and hepatocyte lipid metabolic modulation by strategically transitioning from “stealth mode” to “targeted activation”, ultimately facilitating bidirectional M1/M2 repolarization.

Furthermore, an unconventional macrophage polarization pathway, termed non-classical polarization, has been recognized for its role in bolstering host antibacterial defenses. For instance, implant-associated biofilm infections are predominantly induced by *Staphylococcus aureus*, which establishes biofilms to evade host immune surveillance, culminating in persistent infections and heightened antibiotic resistance [330]. The biofilm-infected microenvironment (BIM), which orchestrates macrophage metabolic reprogramming by downregulating FAO and LDs biogenesis, is an important factor contributing to this process, thereby skewing macrophages toward the immunosuppressive M2 phenotype and attenuating antibacterial immunity. Consequently, Liu et al. [287] designed the nanoplatform HSA-IR820@OA@ZIF-8 (HIROZ), which is capable of modulating macrophage lipid metabolism, while concurrently activating antibacterial immune responses and dismantling biofilm architecture. HIROZ is fabricated via the encapsulation of the near-infrared-II (NIR-II) fluorescence probe Human Serum Albumin-IR820 (HSA-IR820) with oleic acid, followed by co-assembly with the zeolitic imidazolate framework-8 (ZIF-8). In the pathological BIM setting, macrophages display suppressed lipid synthesis pathways, upregulated M2 macrophage markers, and attenuated phagocytic activity. However, HIROZ primarily triggers the release of zinc ions upon NIR-II photothermal activation, causing a surge in bacterial ROS production and membrane destabilization, ultimately resulting in bacterial eradication and the reinstatement of the pro-inflammatory M1 macrophage polarization. HIROZ delivers OA, promoting LDs formation, where LDs surfaces accumulate cathelicidin antimicrobial peptide, directly eliminating intracellular bacteria. This study underscores the critical role of macrophage lipid metabolism as a frontline defense against infections. Hence, lipid metabolic reprogramming strategies, synergized with immune modulation, offer a promising strategy to tackle antibiotic-resistant infections. Notably, compared to conventional antibiotics, HIROZ disrupts biofilm integrity and mitigates recurrence risks, whereas long-term zinc ion accumulation and its potential safety implications warrant further exploration.

In addition to the aforementioned strategies, Kroemer et al. [331] proposed an innovative approach for lipid metabolism reprogramming by concurrently reprogramming the lipid metabolism in tumor and immune cells. Specifically, ICD, a distinct mode of cell demise, is instrumental in activating adaptive immune responses, thereby potentiating antitumor immune responses, inducing durable immune memory, and reinforcing immune surveillance and defense mechanisms. However, ICD-based tumor immunotherapy remains constrained by several challenges. For instance, tumor cells can counteract ICD-triggered LPO via compensatory antioxidant defense mechanisms, such as the GPX4 and ferroptosis suppressor protein 1 (FSP1) pathways. Moreover, immunosuppressive cells within the TME perpetuate immune suppression by exploiting dysregulated lipid metabolism, thus impairing antitumor immune responses. Therefore, Ma et al. [288] implemented a dual-targeted intervention strategy: 1) they modulated tumor lipid metabolism by concurrently suppressing GPX4 and FSP1, thereby amplifying LPO and potentiating ICD; and 2) they reprogrammed immune lipid metabolism to alleviate immunosuppressive constraints. Hollow mesoporous CuS NPs possess intrinsic GPX4-inhibitory activity. Thus, CuS NPs loaded with an FSP1 inhibitor disrupt 2 complementary redox defense pathways and, in synergy with NIR irradiation, potentiate ICD. CuS NPs were coated with 4T1 tumor cell membranes to improve tumor-specific homotypic recognition and minimize off-target cytotoxicity. Based on this foundation, a hydrogel-based delivery platform was engineered, which was designed to co-deliver tumor-targeting CuS NPs and the immune-suppressive cell-targeting CD36 inhibitor sulfo-N-succinimidyl oleate (SSO), ultimately forming iF-CuS-M/SSO@Gel. SSO inhibits lipid uptake in M2-TAMs and MDSCs in vitro and in vivo models, suppresses FAO activity, and facilitates their reprogramming into immune-activated phenotypes, resulting in a reduced Treg cell population. Generally, in terms of antitumor efficacy, the composite hydrogel provided threefold therapeutic benefits: immune activation, tumor suppression, and mitigation of postoperative recurrence. Consequently, this nanoplatform leverages multi-component synergy with precisely controlled release, orchestrating dual metabolic reprogramming across tumor cells and the immune landscape, thereby proposing a novel immunometabolic paradigm for lipid balance modulation in the TME. The core promise of lipid metabolism-reprogramming therapies that act synergistically on both tumor and immune cells lies in their ability to simultaneously deplete the tumor’s “fuel reservoir” and release its “immune brakes”, achieving a “two birds with one stone” synergistic effect. In the future, however, considering that the same metabolic target may exert opposite effects on tumors and immune cells, careful evaluation of the net outcome will be essential.

Furthermore, strategies for targeting lipid metabolic reprogramming have also inspired researchers to explore novel biomaterials suited for different lipid metabolism environments. For instance, nanomaterials, upon contact with bodily fluids, interact with biomolecules, resulting in the formation of a biomolecular corona primarily composed of LDL [332]. Given that LDL functions as a carrier for cholesterol and lipids, structural alterations in LDL may result in excessive macrophage uptake, leading to LDs accumulation and foam cell formation, potentially causing lipid metabolism imbalances [333]. Wu et al. [334] employed graphene oxide (GOs) as a model material to elucidate this mechanism and demonstrated that the hydrophilic and hydrophobic properties of GOs govern LDL structural integrity and functionality, thereby mediating cellular metabolic reprogramming, including LDL recognition, uptake, hydrolysis, efflux, and LDs formation. Specifically, the hydrophobic GOs exhibited a strong affinity for the lipid moieties of LDL, compromising LDL structural stability and triggering LDL aggregation and fusion. Additionally, the hydrophilic GOs preferentially interacted with LDL apolipoproteins, modulating their secondary conformation via electrostatic interactions and impairing LDLR binding domains. Moreover, the hydrophobic GOs/LDL complexes were preferentially internalized by macrophages through sCD36, facilitating LDs accumulation and foam cell formation. Conversely, low internalization efficiency mitigated LDs deposition. The continuous discovery of novel biomaterials has elevated nanomedicine from a passive “cargo carrier” to an active “cellular signal commander”. In the future, integrating such bioactive nanomaterials with traditional drug delivery functions may yield therapeutic effects far superior to those of monotherapies. However, the manufacturing processes, quality control, and standardization of nanomedicines remain major challenges for clinical translation.

## Conclusions and outlook

Lipid metabolism operates through intricately interwoven metabolic networks, where different types of lipid metabolism-related disorders may require treatment of different targets. However, targeting multiple targets simultaneously may have cross-cutting effects [258]. In this regard, advancements in nano-delivery systems have further expanded the flexibility in modulating lipid metabolism, thereby enhancing therapeutic precision and efficacy. In this study, we reviewed the latest advances and representative applications of these nano-delivery platforms with an entry point for interventions targeting various processes involved in lipid metabolism. Lipid metabolism-based drug delivery strategies operate as a “Trojan horse”, harnessing endogenous metabolic pathways of target cells. In summary, most research approaches can be generalized into 2 steps. Initially, crucial regulators, including genes, proteins, enzymes, and metabolic pathways involved in lipid metabolism, are identified as therapeutic targets. Subsequently, targeted delivery platforms are tailored to specific microenvironmental conditions, facilitating the administration of inhibitors, gene-editing tools, or drugs, culminating in multi-level modulation of lipid metabolic processes. In contrast, studies, such as Chen et al. [269], employed an alternative approach by conducting metabolomic analysis to identify differentially expressed lipid metabolites. Their findings demonstrated that restoring the metabolite matairesinol significantly downregulated lipid synthesis-related genes *PNLIP* and *DGAT2*.

Although the abundance of lipid metabolism targets provides extensive regulatory flexibility, it also poses several challenges and limitations. Notably, current research has largely overlooked the complex interactions among metabolic targets. Given that lipid metabolic pathways exhibit intrinsic metabolic plasticity, over-targeting a single pathway may accordingly activate metabolic disruptions in alternative pathways, compromising network stability and overall lipid homeostasis [335]. For instance, prolonged inhibition of FA synthesis may disrupt cholesterol metabolism, potentially increasing the risk of AS and complicating disease management [336]. Moreover, lipid metabolic pathways are often compensatory and exhibit plasticity; therefore, when a target is inhibited or activated, the body may compensate for this change via other pathways, which may lead to diminished therapeutic efficacy or rebound of the disease, creating a rebound effect [337]. In contrast, over extended treatment durations, patients may develop drug resistance, or compensatory metabolic adaptations may gradually attenuate drug efficacy [338]. Furthermore, prolonged administration of lipid metabolism-targeting agents may impair normal immune cell and hepatic function, resulting in metabolic disturbances and immune-related adverse effects [7]. Subsequently, safety concerns remain a major bottleneck restricting the clinical translation of current research. Notably, patient-specific variability has also been overlooked. Lipid metabolism is governed by multifactorial determinants, including genetic predisposition, environmental factors, and lifestyle influences, which contribute to substantial differences in therapeutic responses. Therefore, genetic diversity, individual drug metabolism variations, and dietary habits may significantly affect treatment outcomes, thereby adding complexity to therapeutic strategies.

Therefore, several critical avenues deserve further exploration to address the current limitations of nanocarrier-based therapies for lipid metabolism-related diseases. For instance, a fundamental consideration is that lipid metabolism operates as a finely tuned and dynamic balance. However, focusing on a single therapeutic target may exert only a marginal influence on disease modulation. As a result, firstly, deeper mechanistic insights into the complex crosstalk between lipid metabolism and systemic homeostasis are essential.

Secondly, LPO and ferroptosis, as important and interrelated biological events in lipid metabolism, should be prioritized in the rational design of next-generation nanocarriers. In this regard, engineering nanoplatforms to mimic ferroptotic mechanisms or actively induce ferroptosis offers a promising strategy for inducing targeted cell death [339]. LPO is defined as an oxidative process in which lipid molecules, particularly PUFAs, are oxidized to generate peroxides and constitutes a core mechanism of ferroptosis. Excessive iron ions catalyze LPO, and the resulting peroxidation products compromise membrane integrity, ultimately leading to cell death [340]. Moreover, iron is indispensable in lipid synthesis and metabolism, particularly within organs such as the liver, where it regulates FA synthesis and oxidative pathways. Thus, iron-dependent enzymes, including FASN, play a fundamental role in lipid metabolism [341]. Therefore, recent studies have begun integrating lipid metabolism with ferroptosis, highlighting their therapeutic synergy in cancer treatment. For instance, Wu et al. [342] noted that the highly saturated lipid barrier of tumor cell membranes impedes the propagation of lipid peroxides, thereby constraining ferroptotic susceptibility. They developed a binary lipid nano-regulator through rational molecular design. This system co-delivers a GPX4 inhibitor (Ras-selective lethal 3, RSL3) and a FASN inhibitor (orlistat), facilitating the autonomous co-assembly of both agents via a nano-precipitation method, circumventing the reliance on traditional carrier materials. RSL3 inhibits GPX4, leading to ROS accumulation, while orlistat inhibits FASN, increasing PUFA content. Together, these actions concomitantly disrupt the membrane lipid barrier, intensifying the LPO cascade and overcoming the ultimate resistance to ferroptosis. Moreover, pyroptosis, another form of programmed cell death, is mediated through intracellular inflammasome activation and caspase-1/-11-mediated pathways, leading to the release of pro-inflammatory cytokines such as IL-1β and IL-18. During LPO, PUFAs yield LPO byproducts, which compromise membrane integrity while concurrently activating the NLRP3 inflammasome, thereby triggering pyroptosis. In oxidative stress and LPO environments, this process heightens the susceptibility to inflammation-driven cell death [343]. Given this, recent studies have explored exploiting FA metabolism dysregulation to induce tumor cell pyroptosis. Zhou et al. [344] developed homomultivalent polymeric nanotraps, capable of encapsulating lipid metabolism inhibitors (such as FASN inhibitors or SREBP pathway antagonists). These nanotraps inhibit FA synthesis and cholesterol assimilation, leading to excessive LPO and ROS accumulation. Through precise modulation of key lipid metabolic regulators, this strategy disrupts metabolic homeostasis and induces pyroptosis, culminating in a synergistic anti-tumor effect. Notably, this approach holds promise for application in other malignancies that are highly dependent on lipid metabolism.

Thirdly, from a biomimetic perspective, engineering adipocytes or LDs has emerged as a novel research direction, particularly with the rapid advancement of gene-editing technologies. Previous studies have attempted to modulate adipocyte metabolism and functional pathways through environmental stimuli or cellular reprogramming [345, 346]. The rise of gene-editing technology has introduced numerous innovative advantages in drug delivery applications. By modifying the surface properties of drug delivery carriers, gene editing enables precise targeting of specific genes or genomic regions within target cells or tissues. This high specificity minimizes off-target drug distribution in healthy tissues, thereby reducing toxicity and adverse effects [347]. Additionally, gene editing allows for permanent or long-term genomic modifications in target cells, facilitating sustained drug release and prolonged therapeutic efficacy [348]. More notably, gene-editing technology presents opportunities for personalized medicine in drug delivery. Given the genetic heterogeneity among individuals, gene-editing-based approaches can be customized to match patient-specific genomic characteristics, effectively addressing the challenges of interindividual variability in lipid metabolism therapies [349]. Nguyen et al. [350] applied CRISPRa technology to upregulate the expression of UCP1, PPARGC1A, or PRDM16 genes in adipocytes, thereby enhancing their metabolic activity. The engineered adipocytes were co-cultured with various cancer cells, and the results revealed a significant reduction in cancer cell proliferation. Moreover, cancer cells co-cultured with CRISPRa-modified adipocytes exhibited decreased FA uptake and reduced expression of FAO-related genes such as CD36 and CPT1B. These findings indicate that CRISPRa-modified adipocytes suppress cancer cell growth and metabolism by enhancing metabolic activity.

In summary, there is still great promise in the direction of improving lipid metabolism through drug delivery techniques. Research on NPs and liposomes has attained considerable progress. As biomaterials and gene-editing technologies continue to evolve, it is anticipated that future innovations will facilitate customized therapeutic regimens tailored to individual lipid metabolic responses and genetic differences. Moreover, continuous mechanistic investigations into lipid metabolism targets will also contribute to the advancement of targeted therapeutic strategies, ultimately fostering significant benefits for human health.

## Abbreviations

2-AG: 2-Arachidonoylglycerol
ABCA1: ATP-binding cassette transporter A1
ACAT: Acyl coenzyme A-cholesterol acyltransferase
ACC: Acetyl-CoA carboxylase
ACLY: ATP-citrate lyase
ACSS: Acetyl-CoA synthetase
AD: Alzheimer’s disease
ADPN: Adiponectin
AIE: Aggregation-induced emission
Akt: Also known as protein kinase B, PKB
AMPK: Adenosine monophosphate-activated protein kinase
ATGL: Adipose triglyceride lipase
Aur: Auranofin
AVA: Avasimibe
AS: Atherosclerosis
CD36: Cluster of differentiation 36
CDCA: Controlled-release chenodeoxycholic acid-loaded
COD: Cholesterol oxidase
CRC: Colorectal cancer
CS: Chondroitin sulfate
CS-BR: Chondroitin sulfate-bilirubin
CtBP2: C-terminal binding protein 2
CuS NPs: CuS nanoparticles
CPT: Carnitine palmitoyl transferase
CVDs: Cardiovascular diseases
CRISPR: Clustered regularly interspaced short palindromic repeats
Cas9: CRISPR-associated protein 9
DCs: Dendritic cells
DNL: De novo lipogenesis
DGAT: Diacylglycerol acyltransferase
DHHC: Asp-His-His-Cys
EMT: Epithelial-mesenchymal transition
ER: Endoplasmic reticulum
FABPs: Fatty acid-binding proteins
FAO: Fatty acid β-oxidation
FAs: Fatty acids
FASN: Fatty acid synthase
FATPs: Fatty acid transport proteins
FFAs: Free fatty acids
FSP1: Ferroptosis suppressor protein 1
FU: Fucoidan
FX: Fucoxanthin
GPX4: Glutathione peroxidase 4
GSH: Glutathione
GOs: Graphene oxide
HDL-C: High-density lipoprotein-cholesterol
HMG-CoA: 3-Hydroxy-3-methylglutaryl-CoA
HMGCR: 3-Hydroxy-3-methylglutaryl (HMG)-CoA reductase
HSL: Hormone-sensitive lipase
HSCs: Hepatic stellate cells
HIROZ: HSA-IR820@OA@ZIF-8
ICD: Immunogenic cell death
IPP: Isopentenyl pyrophosphate
IRTM: Immunosuppressive residual tumor microenvironment
LCAD: Long-chain acyl-CoA dehydrogenase
LCF: Licofelone
LCFAs: Long-chain fatty acids
LDs: Lipid droplets
LDL-C: Low-density lipoprotein-cholesterol
LDLR: Low-density lipoprotein receptor
LSECs: Liver sinusoidal endothelial cells
LPO: Lipid peroxidation
LXR: Liver X receptors
mTOR: Mammalian target of rapamycin
MUFA: Monounsaturated fatty acids
MONDs: Molybdenum oxide nanodots
MGLL: Monoglyceride lipase
MYC: Myelocytomatosis virus oncogene cellular homolog
MCAD: Medium-chain acyl-CoA dehydrogenase
M1: Pro-inflammatory phenotype
M2: Anti-inflammatory (M2) phenotype
NADPH: Nicotinamide adenine dinucleotide phosphate hydrogen
NAFLD: Nonalcoholic fatty liver disease
NIR-II: Near-infrared-II
NPs: Nanoparticles
NPC1L1: Niemann-Pick type C1-like 1
Nrf2: Nuclear factor erythroid 2-related factor 2
OA: Osteoarthritis
OMs: Outer membranes
PA: Palmitic acid
PCBP: Pd/hCeO_2_-BMS309403@platelet membrane
PCSK9: Proprotein convertase subtilisin/kexin type 9
PD: Parkinson’s disease
pDox: Doxorubicin prodrug
PDT: Photodynamic therapy
P-G3: Polycation-based nanomedicine polyamidoamine generation 3
PI3K: Phosphoinositide 3-kinase
PLIN: Perilipins
Plu: PluriSin
PMeTPP-MBT: 3,6-bis(4-methylthiophen-2-yl)-2,5-bis(2-octyldodecyl) pyrrolo[3,4-c]pyrrole- 1,4(2H,5H)-dione (PMeTPP) and 3,3′-dimethoxy-2,2′-bithiophene (MBT)
PPa: Phosphorbide A
PPARs: Peroxisome proliferator-activated receptors
PUFAs: Polyunsaturated fatty acids
RA: Rumenic acid
RBCM: Red blood cell membrane
Rec-tNVs: Reconstructed turmeric-derived nanovesicles
ROS: Reactive oxygen species
RSL3: Ras-selective lethal 3
SCAP: SREBP cleavage-activating protein
SCD: Stearoyl-CoA desaturase
SERAC1: Serine active site containing 1
SFAs: Saturated fatty acids
SIB: Silybin
SIM: Simvastatin
siRNA: Small interfering RNA
SQLE: Squalene epoxidase
SREBP: Sterol regulatory element-binding protein
STAT: Signal transducer and activator of transcription
TADCs: Tumor-associated dendritic cells
TAK1: Transforming growth factor-β-activated kinase 1
TAMs: Tumor-associated macrophages
TCA: Tricarboxylic acid
TG: Triglycerides
TME: Tumor microenvironment
TNBC: Triple-negative breast cancer
TOFA: 5-(Tetradecyloxy)-2-furoic acid
Treg: Regulatory T cell
UCP1: Uncoupling protein 1
USP22: Deubiquitinating enzyme ubiquitin-specific protease 22
ZIF-8: Zeolitic imidazolate framework-8
